# Replication catastrophe induced by cyclic hypoxia leads to increased APOBEC3B activity

**DOI:** 10.1093/nar/gkab551

**Published:** 2021-07-01

**Authors:** Samuel B Bader, Tiffany S Ma, Charlotte J Simpson, Jiachen Liang, Sakura Eri B Maezono, Monica M Olcina, Francesca M Buffa, Ester M Hammond

**Affiliations:** Oxford Institute for Radiation Oncology, Department of Oncology, The University of Oxford, Oxford, OX3 7DQ, UK; Oxford Institute for Radiation Oncology, Department of Oncology, The University of Oxford, Oxford, OX3 7DQ, UK; Oxford Institute for Radiation Oncology, Department of Oncology, The University of Oxford, Oxford, OX3 7DQ, UK; Oxford Institute for Radiation Oncology, Department of Oncology, The University of Oxford, Oxford, OX3 7DQ, UK; Oxford Institute for Radiation Oncology, Department of Oncology, The University of Oxford, Oxford, OX3 7DQ, UK; Oxford Institute for Radiation Oncology, Department of Oncology, The University of Oxford, Oxford, OX3 7DQ, UK; Oxford Institute for Radiation Oncology, Department of Oncology, The University of Oxford, Oxford, OX3 7DQ, UK; Oxford Institute for Radiation Oncology, Department of Oncology, The University of Oxford, Oxford, OX3 7DQ, UK

## Abstract

Tumor heterogeneity includes variable and fluctuating oxygen concentrations, which result in the accumulation of hypoxic regions in most solid tumors. Tumor hypoxia leads to increased therapy resistance and has been linked to genomic instability. Here, we tested the hypothesis that exposure to levels of hypoxia that cause replication stress could increase APOBEC activity and the accumulation of APOBEC-mediated mutations. APOBEC-dependent mutational signatures have been well-characterized, although the physiological conditions which underpin them have not been described. We demonstrate that fluctuating/cyclic hypoxic conditions which lead to replication catastrophe induce the expression and activity of APOBEC3B. In contrast, stable/chronic hypoxic conditions which induce replication stress in the absence of DNA damage are not sufficient to induce APOBEC3B. Most importantly, the number of APOBEC-mediated mutations in patient tumors correlated with a hypoxia signature. Together, our data support the conclusion that hypoxia-induced replication catastrophe drives genomic instability in tumors, specifically through increasing the activity of APOBEC3B.

## INTRODUCTION

Hypoxia, which is defined as conditions of insufficient oxygen, is associated with the resistance of tumors to standard therapies and poor patient prognosis (1,2). Tumor hypoxia has been described as chronic or acute but is also cyclic/intermittent. Regions of chronic hypoxia occur at distances beyond the diffusion distance of oxygen in tissue, whilst acute hypoxia can occur near to vessels as a result of the inefficient and malformed vasculature. Cyclic or intermittent hypoxia in tumors is characterized by fluctuating oxygen levels due to both angiogenesis and red blood cell flux (3–6).

The extreme levels of hypoxia (<0.1% O_2_) most associated with therapy resistance, referred to as radiobiological hypoxia, lead to replication stress (7). Replication stress is highly complex and can be broadly defined as situations where DNA replication fork dynamics are abnormal. Our studies have shown that radiobiological hypoxia induced replication stress, which includes the rapid accumulation of single stranded DNA (ssDNA), leads to a DNA damage response (DDR) (8). Importantly, radiobiological hypoxia is unusual in that the induction of replication stress does not lead to an apparent accumulation of DNA damage, which suggests that the level of replication stress is transient or mitigated by other factors (9,10). This lack of associated DNA damage observed in hypoxia, even after prolonged exposures, might indicate that despite a robust replication stress response, damage is avoided potentially by reduced DNA replication/transcription rates (10,11). In contrast, a number of commonly used pharmacological agents, which lead to replication fork stalling, have been shown to lead to DNA damage and this has been attributed to RPA exhaustion resulting in replication catastrophe (12).

The apolipoprotein B mRNA-editing enzyme, catalytic polypeptide-like (APOBEC) proteins are a family of conserved cytidine deaminases that play a critical role in viral immunity (13,14). APOBEC-mediated cytosine deamination to uracil can occur in RNA but primarily occurs in regions of ssDNA including at R-loops, stalled replication forks, transcription bubbles, and during end resection of damaged DNA (15–19). Many C to T and C to G mutations within the APOBEC consensus deamination sequence (TCW) have been observed in the genomic sequences of patient tumors and attributed to APOBEC enzymatic activity. This observation has led to the characterization of an APOBEC mutational signature (20,21). The frequency of the APOBEC signature in cancers is second only to the ageing signature and the majority of APOBEC-induced mutations in human cancers have been attributed to either APOBEC3A (A3A) or 3B (A3B) expression (20–24). Notably, A3B activity has been shown to increase in response to hydroxyurea (HU)-induced replication stress in an ATR-dependent manner (25). The current paradigm is that cancers where this mutational signature predominates have deregulated APOBEC activity, and this contributes to cancer progression by increasing genomic instability and treatment resistance through an elevated mutation rate. Correspondingly, an increase in A3B expression can be seen in many of the cancers that possess an APOBEC mutational signature (22,26). In contrast to the pro-tumorigenic consequences of A3B overexpression, it has recently been shown that A3B overexpression can increase the sensitivity of tumor cells to immune checkpoint blockade therapy through an increase in the presentation of novel neoepitopes on the surface of cancer cells (27). It is currently unclear whether increasing or ablating APOBEC activity in tumors would be beneficial therapeutically. Importantly, the mechanism by which A3B initially becomes deregulated in human cancer has not been described. It is crucial that we understand how APOBECs are regulated in tumors and particularly as specific inhibitors become available. Hypoxia-induced replication stress, which includes the significant accumulation of the APOBEC substrate, ssDNA, suggests that hypoxia could be a critical factor in regulating APOBEC expression and activity in tumors.

## MATERIALS AND METHODS

### Cell lines and reagents

Colorectal RKO (ATCC), RKO^HIF1a+/+^ and RKO^HIF1a−/−^ (28), HCT116, Breast MCF7, MDA-MB231, Bladder T24, VMCUB1 (provided by Prof Anne Kiltie, Oxford University) and HCT116 p53+/+ and p53−/− (provided by Prof Bert Vogelstein, Johns Hopkins Medicine) cells were grown in DMEM media. Non-tumorigenic breast MCF10A (provided by Prof Paul Span, Nijmegen University) cells were grown in MEGM media. Non-transformed lung MRC5 cells (ATCC) were grown in EMEM media. Esophageal OE21 cells (PHE) were grown in RPMI media. All cell media was supplemented with 10% FBS, except MEGM media which was supplemented with 20% FBS, and cells were maintained in an incubator set at 37°C and 5% CO_2_. All cell lines were verified mycoplasma free using a HEK-Blue™ detection kit (Invivogen). Inhibitors/drugs used were: VX-970 (M6620) (MedChemExpress), AZD6738 (MedChemExpress), Gö6976 (Sigma Aldrich), MK-8776 (Selleckchem), Bay11-7085 (Tocris Biosciences), PHA-767491 (Selleckchem), *N*’ acetylcysteine (NAC) (Sigma Aldrich), and roscovitine (Ros) (Selleckchem). For siRNA-mediated knockdowns, RKO cells were transfected with siRNA to a final concentration of 50 nM using DharmaFECT 1 (T-2001-03; Horizon Discovery, Cambridge, UK) following manufacturer's protocols. Sequences of siRNAs used: siA3B (CCUGAUGGAUCCAGACACA[dT][dT]), sip53 (GUAAUCUACUGGGACGGAA[dT][dT]), and siATR (CAG GCA CTA ATT GTT CTT CAAd[T]d[T]).

### Hypoxia exposure

Hypoxia treatments at <0.1% O_2_ were carried out in a Bactron II anaerobic chamber (Shel Labs) while all cyclic treatments were carried out in an M35 variable atmosphere workstation (Don Whitley Scientific). Oxygen concentrations were periodically validated using anaerobic oxygen indicator strips (Thermo Fisher) and an Oxylite oxygen probe (Oxford Optronix). Cells were seeded on glass dishes and harvested in the chamber with equilibrated solutions.

### RT-qPCR

Total RNA was isolated from cells with Trizol (Invitrogen/Life Technologies) and was reverse transcribed into cDNA with Verso enzyme kit (Thermo Scientific). SYBR Green PCR Master Mix kit (Applied Biosystems) was mixed with gene specific primers and the reaction was run on a StepOne Real-Time PCR System (Thermo Scientific). Fold change values relative to normoxia (21% O_2_) were determined using the ΔΔct method using 18S as an endogenous control. Graphs were plotted using the mean of three biological replicates ± SEM. Primers sequences are given in Supplementary Table S1.

### Deamination assay

Cells were lysed in HED lysis buffer (25 mM HEPES, 5 mM EDTA, 10% glycerol, 0.2% NP-40, 1 mM DTT (added fresh), 1X protease inhibitor (Roche) (added fresh), homogenized with a 25 G needle, and sonicated briefly in a Biorupter plus sonicator (Diagenode) before pre-clearing by centrifugation for 10 min. A deamination mix was made consisting of 8.25 ml of 5 μg/μl protein lysate, 0.625 μl RNaseA, 0.625 μl TE buffer, and 0.5 μl of 200 μM ssDNA oligo (5′-ATTATTATTANTCAAATGGATTTATTTATTTATTTATTTATTT’FAM3′) and incubated at 37°C for 3 h. Samples were then treated with 100 mM NaOH at 95°C for 30 min. Samples were mixed with Gel loading buffer II (Invitrogen) and incubated at 70°C for 3 min before being run on a 15% TBE-urea gel (Novex). Bands were analyzed with a Chemi-doc touch imaging system (Bio-Rad). Product bands were quantified using the analyze gels tool in ImageJ.

### Western blotting

Cells were lysed in HED lysis buffer and treated as with the deamination assay. Proteins were separated on a 4–20% polyacrylamide gel (Bio-Rad) and blotted onto a nitrocellulose membrane (Bio-Rad). Bands were imaged using Odyssey Infrared Imaging (LI-COR Biosciences). Experiments were carried out in triplicate and a representative blot is shown for each experiment. Antibodies used; Cell Signaling: p53-S15 (9284), RPA32 (2208), Chk1-S345 (2341), Chk1-S296 (90178), H3 (3638), H3-S10 (9701), p65 (3033), p65-S536 (93H1), cGAS (15102); Santa Cruz: p53 (sc-126), β-Actin (sc-69879), Chk1 (sc-8408), ATR (sc-515173); Bethyl: KAP1 (A300-274A), KAP1-S824 (A300-767A); Millipore: γH2AX (05–636), H2AX (07–627); Abcam: A3B (ab184990), Cyclin A (ab181591); BD Biosciences: HIF-1α (610958), GRP78 (610979).

### Fluorescence activated cell sorting (FACS)

For BrdU incorporation plots, freshly made up BrdU (20 μM) was added in the dark to cells for 1 h before fixation in ice-cold 70% ethanol. Samples were treated with 2 M HCl and blocked in 2% FBS PBS solution. Propidium Iodide (Sigma Aldrich) was added to samples to determine total DNA content. Antibodies used were: BrdU (BD biosciences #347580), H3-S10 (Cell Signaling #9701) and Alexa Fluoro 488 (Invitrogen). Samples were run on a BD FACSCaliber machine (BD biosciences) and plots were analyzed with FloJo software.

### Immunofluorescence

Cells were seeded onto autoclaved cover slips (Menzel-Glaser) before treatment. Cells were fixed with 4% fixation buffer (4% (w/v) paraformaldehyde in PBS). Samples were permeabilized with 1% PBS-Triton X-100 (Thermo Fisher) and blocked in 2% (w/v) BSA (Thermo Fisher) in 0.1% PBS-tween. For A3B staining, samples were permeabilized with 0.1% PBS–Triton X-100 (Thermo Fisher) and blocked in 5% (w/v) BSA (Thermo Fisher) in PBS. For EdU staining, cells were incubated with EdU (10 μM) (Thermo Fisher) and Click-iT Alexa Fluor 657 labelling kit (Thermo Fisher) was used for staining. Proteins were visualized with an LSM710 confocal microscope (Carl Zeiss Microscopy Ltd) and at least 150 cells were counted per condition. DNA damage and replication stress were quantified by counting cells with >5 53BP1 foci or >6 RPA foci respectively. Antibodies used: 53BP1 (Novus Biologicals #NB100-904), A3B (Origene TA349029), RPA (Cell Signaling #2208), RPA-S4/S8 (Abcam ab243866), Alexa Fluoro 488 (Invitrogen), and Alexa Fluoro 594 (Invitrogen).

### TCGA data analysis

Raw expression data from 51 genes within a validated hypoxia signature (29) and data from 6 hypoxia inducible p53 response genes (BTG2, CYFIP2, INPP5D, KANK3, PHLDA3 and SULF2) (30) were taken from TCGA datasets downloaded from the GDC data portal (https://portal.gdc.cancer.gov/) to represent hypsig and p53genes respectively. The harmonized TCGA data were considered (31). The mean value of hypsig and p53genes within a tumor sample was then calculated as a summary score of their expression (32). Mutational data for TCGA datasets considered in this study were obtained from a previous study (21). Datasets were selected where there were >100 samples with available mutational data, these were BRCA and LUAD (21). APOBEC mutations were defined as the number of C to T or C to G mutations occurring in the TCW sequence context in a given sample. Mutation values were logged base 10 and shifted by 0.5 for visual reason so to display data from tumors with no observed APOBEC mutations. Summary scores for expression signatures, APOBEC gene expression and the number of APOBEC mutations contained in tumor samples were correlated using non-parametric Spearman's rank-order correlation test to avoid issues from non-normality of the data distributions and the effect of outliers.

### Other statistical analysis

A two-tailed, paired Student's *t*-test was used for the comparison of two means and a one-way analysis of variance (ANOVA) was used for the comparison of more than two means. Statistical significance was assumed if *P* <0.05. Error bars represent mean ± standard error of the mean (SEM).

## RESULTS

### Hypoxia-induced replication stress leads to increased APOBEC expression but decreased deamination

**Figure 1.**
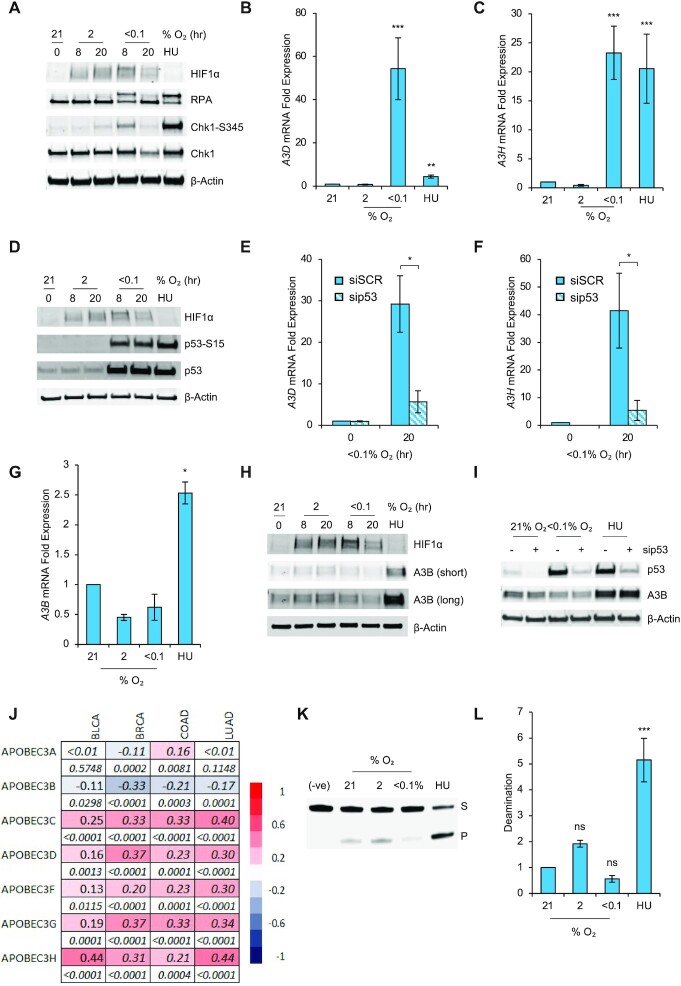
Hypoxia-induced replication stress leads to increased APOBEC expression but decreased deamination. (**A**) RKO cells were exposed to the indicated oxygen concentration or HU (2 mM, 20 h) for the indicated times and western blotting was carried out; β-actin was used as a loading control. (**B, C**) RKO cells were exposed to the oxygen concentrations indicated or HU (2 mM) for 20 h. The mRNA levels of the indicated APOBECs, *A3D* (B) and *A3H* (C), as a relative fold change to normoxic (21% O_2_) levels are shown. (**D**) RKO cells were exposed to the oxygen concentrations indicated for the times shown or HU (2 mM, 20 h) followed by western blotting as indicated. (**E, F**) RKO cells were treated with siRNA to p53 (sip53), or a scramble control (siSCR) followed by exposure to <0.1% O_2_ for 20 h. mRNA levels of *A3D* (E) and *A3H* (F) are shown relative to the normoxic control. (**G**) RKO cells were exposed to the oxygen concentrations indicated for 20 h or HU (2 mM, 20 h). The mRNA level of *A3B* as a relative fold change to normoxic (21% O_2_) level is shown. (**H**) RKO cells were exposed to the conditions indicated or HU (2 mM, 20 h) followed by western blotting. For clarity, long and short exposures of A3B blot are shown. (**I**) RKO cells were treated with siRNA to p53 (sip53) or a scramble control (siSCR) (indicated as -) followed by exposure to the indicated oxygen concentration or HU (2 mM) for 20 h. Western blotting was carried out. (**J**) Spearman rank-order correlation between a signature of hypoxia-induced p53 response genes and APOBEC mRNA expression in Bladder Urothelial Carcinoma (BLCA), Breast Invasive Carcinoma (BRCA), Colorectal Adenocarcinoma (COAD), and Lung Adenocarcinoma (LUAD) from the TCGA harmonized dataset (see methods). For each gene the correlation values (top raw) and *P* values (bottom raw) are given. Color scale of correlation values is shown on the right of the heatmap. (**K**) RKO cells were exposed to the indicated oxygen concentrations or HU (2 mM) for 20 h followed by a deamination assay. The top band (S) is the substrate band and the bottom band (P) is the product band. (**L**) Quantification of data shown in part K where the average intensity of the product band (P) across three biological repeats is plotted relative to the intensity of the normoxic product band. Data from three separate experiments (*n* = 3) are displayed ± standard error of the mean (SEM) unless specified otherwise. *P < 0.05, **P < 0.01, ***P < 0.001.

To confirm the induction of replication stress, RKO cells were exposed to hypoxia (<0.1% O_2_) and changes to RPA and Chk1 were determined. Both RPA and Chk1 phosphorylation increased in hypoxia (<0.1% O_2_) but, as previously described, returned to near normoxic levels after longer exposure (20 hours) (8). Importantly, the induction of replication stress in response to hypoxia was found to be oxygen dependent, with no replication stress observed at 2% O_2_ (Figure 1A). Here, we investigated the APOBEC3 family members and their response to hypoxia. RKO cells were exposed to <0.1 or 2% O_2_ followed by RT-qPCR. HU was used as a positive control to induce APOBEC expression (25) and the levels of the p53 target gene, *INPP5D*, and a target of the unfolded protein response, *CHOP*, were determined to demonstrate an oxygen-dependent hypoxia response (Supplementary Figure S1A, B) (30,33). All APOBEC3 mRNA transcripts except for APOBEC3A (A3A) and APOBEC3G (A3G) could be detected in the RKO cell line. Of the 5 APOBEC3s detected (A3B, APOBEC3D (A3D), APOBEC3H (A3H), APOBEC3F (A3F) and APOBEC3C (A3C)), A3D and A3H were significantly induced in hypoxia in an oxygen-dependent manner (Figure 1B, C). A similar pattern of expression was observed for A3C and A3F although this failed to reach statistical significance (Supplementary Figure S1C, D). To verify that this was not restricted to the RKO cell line we determined the expression of A3H in the MCF-7 cell line and again observed a significant increase in response to hypoxia (Figure S1E). The induction of A3D and A3H at <0.1 but not at 2% O_2_ suggests that the hypoxia induced factor (HIF1) is not involved in APOBEC expression as HIF1 is stabilized at both <0.1 and 2% O_2_. However, to confirm this, RKO cells with and without HIF1α were treated with hypoxia (<0.1% O_2_) and the levels of A3D and A3H determined. Induction of A3D in response to hypoxia was not changed by the lack of HIF1α while the induction of A3H in response to hypoxia increased further. These data demonstrate that neither A3D nor A3H were induced in a HIF1 dependent manner in hypoxia (<0.1% O_2_) (Supplementary Figure S1F, G). Both A3D and A3H have been described previously as targets of the p53 transcription factor, suggesting that hypoxia-induced p53 could transactivate A3D/A3H (34). In support of this, the induction of p53 in hypoxia shows the same oxygen dependency as observed for A3D/A3H induction (Figure 1D). Using siRNA specific for p53, expression of p53 was reduced in normoxic (21% O_2_) and hypoxic (<0.1% O_2_) conditions. Successful p53 knockdown was verified by the decrease in mRNA expression of *INPP5D*, a validated hypoxia-inducible p53 target gene (Supplementary Figure S1H) (30). As expected, in the absence of p53 the hypoxia-induced expression of both A3D and A3H was abrogated, demonstrating they are induced in a p53-dependent manner in hypoxia (<0.1% O_2_) (Figure 1E, F). However, in contrast to A3D, A3H, A3C and A3F, the expression of A3B mRNA did not increase in response to hypoxia (<0.1% O_2_) (Figure 1G). To confirm that the decrease in A3B in response to hypoxia was not restricted to RKO cells, we measured the levels of A3B mRNA in response to hypoxia in seven additional cells lines and saw a reduction in each (Supplementary Figure S1I). Next, we investigated the level of A3B protein in response to hypoxia and found that A3B expression was moderately reduced while a clear induction was observed in HU treated cells (Figure 1H). Previous reports have suggested that p53 represses the expression of A3B mRNA (34,35). Repression of A3B by p53 in hypoxia seemed unlikely as decreased expression of A3B was observed in several p53 mutant cell lines (Supplementary Figure S1I, J). However, to formally verify this we depleted p53 in RKO cells and used the genetically matched p53^+/+^ and p53^–/–^ HCT116 cell lines and demonstrated that loss of p53 had no impact on A3B expression in hypoxic conditions (Figure 1I, Supplementary Figure S1K). The p53-mediated response to hypoxia is both oxygen-dependent and extremely stress specific with only select p53-target genes being induced. We have previously characterized a group of six genes induced in hypoxia in a p53-dependent manner, which showed coherent expression in clinical samples (30), and so asked if there was a correlation between these genes and members of the APOBEC3 family. Using TCGA datasets, specifically, Bladder Urothelial Carcinoma (BLCA), Breast Invasive Carcinoma (BRCA), Colorectal Adenocarcinoma (COAD), and Lung Adenocarcinoma (LUAD), a highly significant positive correlation (*P*≤ 0.0001 in 17 cases, *P*≤ 0.001 in two cases, *P* = 0.01 in one case) was found between A3C, A3D, A3F, A3G, A3H and the mean score of the six hypoxia-induced p53-dependent genes in all these cancers (Figure 1J). At a single gene level, we observed differences in the degree of correlation and loss of correlation for specific genes in specific cancer types. While this is expected, the overall results were similar (Table S2). Interestingly, the expression of A3C and A3F positively correlated with the 6 gene signature, supporting our observation of a trend to increased expression in response to hypoxia *in vitro*, for these two genes (Supplementary Figure S1C, D). As predicted by our *in vitro* data, a negative correlation was seen between A3B and the mean expression of the 6 p53-dependent genes in all datasets, furthering the evidence for the decrease in A3B in hypoxia (<0.1% O_2_). However, as all members of the APOBEC3 family have deamination activity, we hypothesized that deamination could increase in response to replication stress in hypoxia as a result of increased expression of A3C, A3D, A3F and A3H, despite unchanged A3B levels. Using an *in vitro* deamination assay, we observed the expected increase in deamination in response to HU, however deamination was unchanged in both of the hypoxic conditions tested (2 and <0.1% O_2_) (Figure 1K, L). These data suggested that the expression of A3B, which has been reported to be one of the two APOBEC3 proteins primarily responsible for the generation of the APOBEC mutational signature, might also dictate the level of deamination in hypoxic conditions. Most interestingly, these data highlight a difference in the biological response to hypoxia and HU-induced replication stress and demonstrate that APOBEC activity is not induced in response to all conditions which lead to replication stress.

### Hypoxia-induced replication stress is distinct to the response to HU

**Figure 2.**
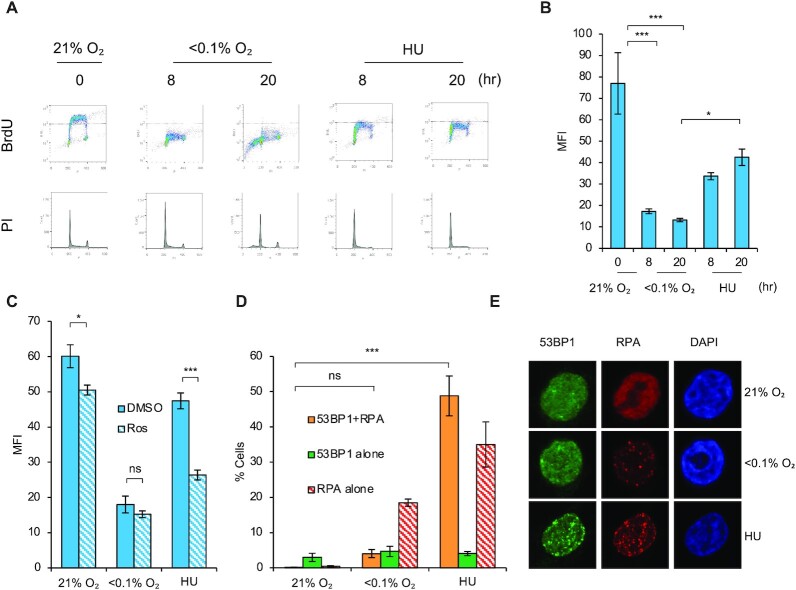
Hypoxia-induced replication stress is distinct to the response to HU. (**A**) RKO cells were exposed to the indicated oxygen concentrations or HU (2 mM) for the indicated times. Cells were labeled with BrdU (20 μM) 1 h prior to collection and analyzed by FACS. (**B**) Quantification of data in A. showing the mean fluorescence intensity (MFI) of cells in each condition. (**C**) RKO cells were pre-treated with DMSO or roscovitine (20 μM) for 30 min and exposed to normoxia (21% O_2_), <0.1% O_2_ or HU (2 mM) for 20 h. Cells were pulsed with BrdU (20 μM) 1 h prior to collection and analyzed by FACS. Mean fluorescence intensity (MFI) in each condition is shown. Exemplar FACs plots are shown in Figure S2C. (**D**) RKO cells were exposed to the indicated oxygen concentration or HU (2 mM) for 20 h. 53BP1 and RPA foci were visualized by immunofluorescence. The percentages of cells with >5 53BP1 and >6 RPA foci are shown. (**E**) Representative images of data shown in part D. Data from three separate experiments (*n* = 3) are displayed ± standard error of the mean (SEM). * *P* < 0.05, *** *P* < 0.001.

To explain the contrasting biological responses to hypoxia and HU, we began with further investigation of the impact of both HU and hypoxia (<0.1% O_2_) on DNA replication. RKO cells were exposed to HU or hypoxia and both propidium iodide (PI) and bromodeoxyuridine (BrdU) incorporation was analyzed (Figure 2A, B and Supplementary Figure S2A). The most noticeable difference between the two treatments was the presence of a sub-G1 population indicative of apoptosis in the hypoxic cells but not the HU treated cells, which was surprising given that the induction of p53 is similar in both cases (Figure 1D, Supplementary Figure S2B). As expected, hypoxia (<0.1% O_2_) lead to a significant decrease in the mean fluorescence intensity (MFI) of the BrdU staining indicating decreased DNA replication. However, it was notable that the HU treated cells were incorporating significantly more BrdU compared to the hypoxic cells (Figure 2B). We predicted that the residual DNA replication in HU treated cells included origin firing, and therefore we used an inhibitor of CDK1, 2, 5 and 7 (roscovitine) to block origin firing and determined the impact on BrdU incorporation. As expected, inhibiting origin firing significantly decreased BrdU incorporation in the HU treated cells compared to control cells (Figure 2C, Supplementary Figure S2C). We hypothesized that on-going replication in HU treated cells would lead to RPA exhaustion and ultimately replication catastrophe (12,36,37). In contrast, we know that hypoxic cells do not experience replication catastrophe, as evidenced by a lack of DNA damage, and attribute this to the extremely limited replication/origin firing (7). To definitively confirm replication catastrophe in response to HU but not hypoxia, we co-stained treated cells for a marker of DNA damage (53BP1) and replication stress (RPA) and measured the proportion of cells that contained both 53BP1 and RPA foci (Figure 2D, E; foci count per cell are also shown Supplementary Figures S2D–F). Together, these data demonstrate that HU-induced replication stress, which leads to APOBEC activity, includes replication catastrophe, while replication catastrophe does not occur in response to hypoxia (<0.1% O_2_).

### Cyclic hypoxia leads to replication catastrophe

**Figure 3.**
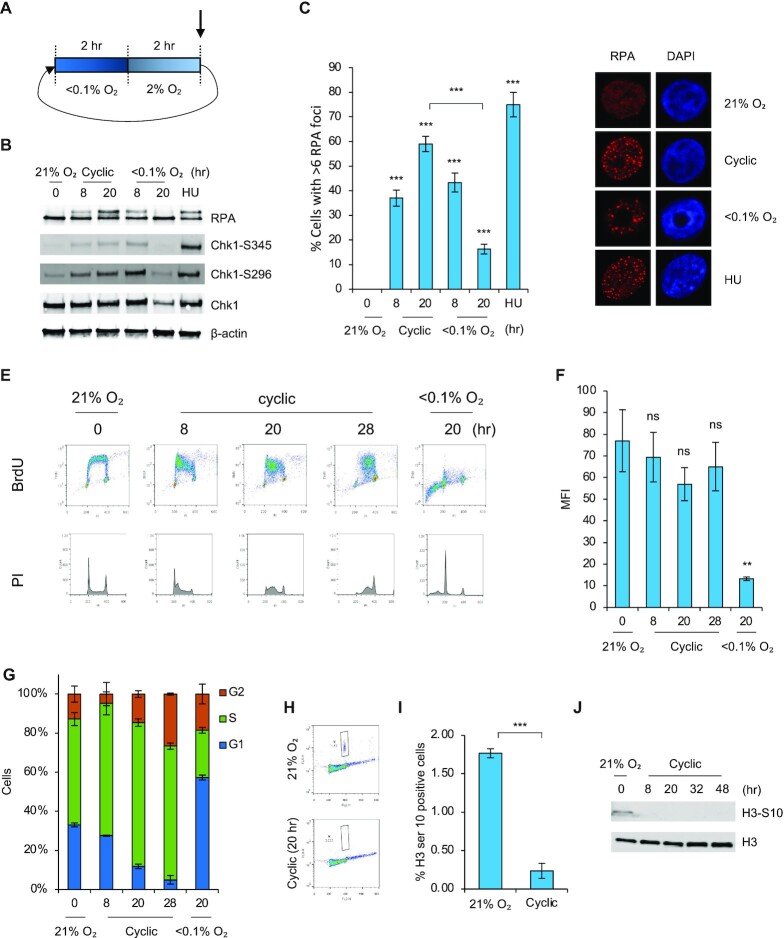
Cyclic hypoxia causes replication stress and a G2 cell cycle arrest. (**A**) A schematic representation of the cyclic hypoxia treatment schedule. Cells were treated with <0.1% O_2_ for 2 h followed by 2% O_2_ for 2 h. This cycle then repeated. The actual O_2_ concentration inside the chamber over a period of 5 cycles is shown in Figure S3A. The arrow indicates the point at which samples were harvested i.e., within the last 5 min of the 2% O_2_ segment. (**B**) RKO cells were exposed to the oxygen concentrations indicated for the times shown or HU (2 mM, 20 h). Western blotting was carried out. (**C**) RPA foci were visualized by immunofluorescence in RKO cells treated with cyclic hypoxia, hypoxia (<0.1% O_2_), or normoxia (21% O_2_) for the indicated times, or HU (2 mM, 20 h). The percentage of cells with >6 RPA foci is shown. (**D**) Representative images from data shown in part C. (**E**) RKO cells were exposed to cyclic hypoxia, hypoxia (<0.1% O_2_), or normoxia (21% O_2_) for the indicated times. Cells were labeled with BrdU (20 μM) 1 h prior to collection and analyzed by FACS. The 21 and <0.1% O_2_ treated conditions are the same data shown in Figure 2A as these experiments were conducted at the same time. (**F**) Quantification of the data in part E showing the MFI of cells in each condition is shown. (**G**) Quantification of the data in part E showing the percentage of cells in each phase of the cell cycle. (**H**) RKO cells were exposed to cyclic hypoxia or normoxia (21% O_2_) for 20 h. FACS was carried out to assess the percentage of cells with H3 ser 10 positive staining. (**I**) Quantification of data shown in H. (**J**) RKO cells were exposed to cyclic hypoxia for the indicated timepoints. Western blotting was carried out. *n* = 1 Data from three separate experiments (*n* = 3) are displayed ± standard error of the mean (SEM) unless specified otherwise. *** *P* < 0.001.

These data led us to refine our original hypothesis and consider that APOBEC activity is induced by replication catastrophe as opposed to replication stress. To test this hypothesis, we considered hypoxic conditions which might include some origin firing and thus could maintain active DNA replication potentially leading to replication catastrophe. Specifically, we asked if including periods of milder hypoxia (2% O_2_), which alone do not induce replication stress, might lead to increased replication and the DNA damage associated with replication catastrophe. Using a hypoxia chamber capable of rapidly switching between multiple oxygen concentrations, we investigated cyclic hypoxia conditions consisting of periods of <0.1% O_2_, which induce replication stress, and 2% O_2_ which leads to HIF stabilization but no replication stress (Figure 3A). The choice of these cycling conditions is further supported by studies which have measured the periodicity and magnitude of oxygen fluctuations in xenograft tumors (38). Measurements of oxygen concentrations were taken using a built-in oxygen probe over the course of all cyclic experiments (Supplementary Figure S3A). We confirmed that the addition of these periods of milder hypoxia (2% O_2_) still led to replication stress by showing that Chk1 and RPA were phosphorylated in cyclic hypoxia (Figure 3B). As seen previously both RPA and Chk1 phosphorylation was reduced after 20 hours in stable hypoxia (<0.1% O_2_), however these phosphorylations appeared to be maintained in cyclic hypoxia. To investigate further, we determined the number of cells with RPA foci in each condition and found that the percentage of cells with RPA foci increased over time in cyclic hypoxia, while in stable hypoxia they formed and decreased over time (Figure 3C, D). Interestingly, when we determined the number of RPA foci per cell, we saw that this number remained constant in cyclic conditions while it decreased over time in stable hypoxia (Supplementary Figure S3B). Together, these data suggested that replication stress persists in cyclic hypoxia compared to stable/chronic conditions. Supportively, cells in cyclic hypoxia incorporated BrdU at a similar rate as those in normoxia, demonstrating on-going DNA replication (Figure 3E, F). An increase in the sub-G1 population was not observed in the cycling conditions in contrast to the stable/chronic hypoxia shown previously (compare Figure 3E to Figure 2A). Interestingly, we found that p53 was phosphorylated and stabilized in cyclic conditions suggesting that while p53 induced in response to stable hypoxia (<0.1% O_2_) leads to apoptosis this is not evident in cycling conditions (Supplementary Figure S3C) (30). Furthermore, cells in cyclic hypoxia accumulated in S-phase before eventually arresting in G2 (Figure 3G). A G2 arrest was confirmed by staining for the absence of the mitotic marker H3 serine 10 detected by both FACS and western blotting (Figure 3H, I, J).

**Figure 4.**
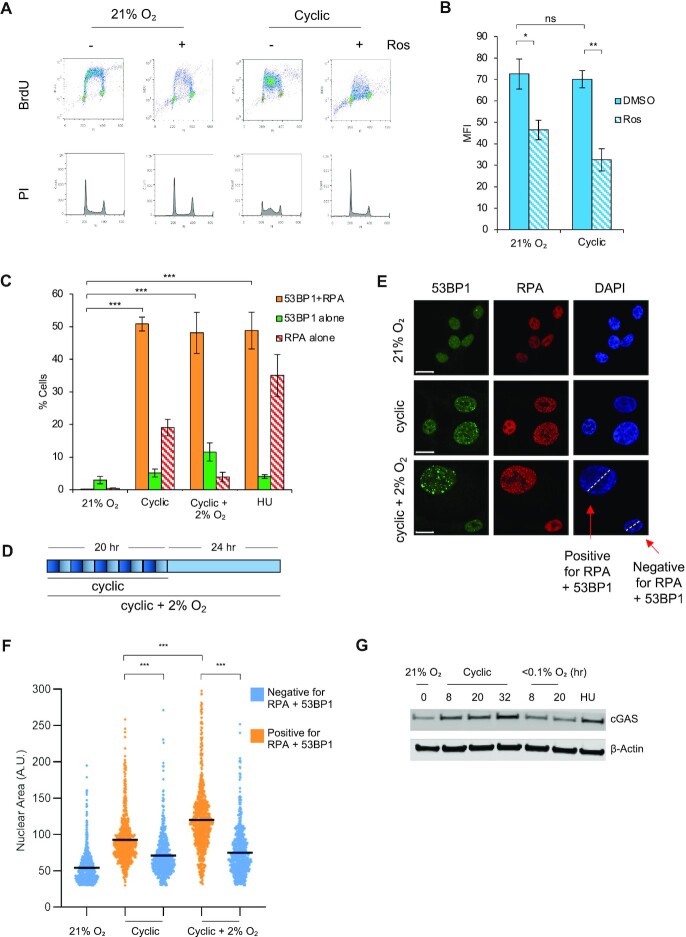
Cyclic hypoxia causes replication catastrophe. (**A**) RKO cells were pretreated with DMSO or roscovitine (Ros) (20 μM) for 30 min and exposed to 21% O_2_ or cyclic hypoxia for 20 h. Cells were labeled with BrdU (20 μM) 1 h prior to collection and analyzed by FACS. (**B**) Quantification of the data in part A showing the mean fluorescence intensity (MFI) in each condition. (**C**) RKO cells were treated with the indicated oxygen treatments or HU (2 mM, 20 h) and co-stained for 53BP1, RPA, and DAPI. Foci were visualized by immunofluorescence. The 21% O_2_ and HU treated conditions are the same data shown in Figure 2D as these experiments were conducted at the same time. (**D**) Schematic representation of the cyclic and cyclic + 2% O_2_ conditions used in part C, E, F. Dark blue represents <0.1% O_2_ and light blue represents 2% O_2_. (**E**) Representative pictures of data in C. The dashed white line demonstrates the difference in cell size. Scale bar = 10 μm. (**F**) Cells were divided into two groups; negative for >5 53BP1 + >6 RPA foci and positive for > 5 53BP1 and >6 RPA. The mean nuclear area of cells in both groups in E were calculated using Fiji software and the nuclear size of individual cells plotted for each condition. (**G**) RKO cells were exposed to the indicated oxygen concentrations for the times shown or HU (2 mM, 20 h), followed by western blotting. Data from three separate experiments (n = 3) are displayed ± standard error of the mean (SEM). * *P* < 0.05, **P < 0.01, ***P < 0.001.

Importantly, the BrdU MFI of cells in cyclic hypoxia decreased when cells were treated with roscovitine, suggesting that on-going replication in these conditions included origin firing (Figure 4A, B). Having characterized the cellular response to cyclic hypoxia, we asked if DNA damage accumulated in the cyclic conditions. We began by co-staining cells in cyclic hypoxia for RPA and γH2AX and determined a significant correlation between these two markers (Supplementary Figure S3D). However, because chronic hypoxia leads to an increase in pan-nuclear γH2AX staining we investigated changes in 53BP1 foci in cyclic hypoxia. Cells exposed to cyclic hypoxia accumulated 53BP1 and this occurred predominantly in the S-phase population (Supplementary Figure S3E). Furthermore, co-staining for 53BP1 and RPA demonstrated that >50% of cells in cyclic hypoxia were positive for both 53BP1 and RPA foci demonstrating that these conditions induced replication catastrophe (Figure 4C). In addition, to determine the kinetics of DNA repair after exposure to cyclic conditions, cells were also exposed to cyclic hypoxia followed by a period at 2% O_2_. Interestingly, when the cyclic hypoxic cells were released into 2% O_2_, a significant number of cells with 53BP1 and RPA foci remained 24 h later (Figure 4C, D). This approach also highlighted that cells retaining DNA damage appeared larger than non-damaged comparators (Figure 4E). Notably, the cells released into 2% O_2_ after cyclic treatment further accumulated DNA damage over time as measured by the absolute number of 53BP1 foci per cell compared to those treated in cyclic alone or with HU (Supplementary Figure S3F–I). Further analysis of cell size determined that the mean nuclear size of cells positive for both RPA and 53BP1 foci i.e. experiencing replication catastrophe, was significantly larger than undamaged cells and this was further increased after reoxygenation to 2% O_2_ (Figure 4F). As an additional measure of DNA damage, the protein expression of the cytoplasmic DNA sensor, cGAS was determined in stable (<0.1% O_2_) and cyclic hypoxia. A robust increase in cGAS protein was seen in response to cyclic hypoxia while no increase was observed in stable hypoxia (Figure 4G). Together, these data are supportive of cyclic hypoxia-inducing replication catastrophe as cells that experience replication catastrophe have been shown to demonstrate persistent replication stress, DNA damage, and an increase in nuclear size after the stress is removed (12).

### Cyclic hypoxia increases the expression and activity of A3B

**Figure 5.**
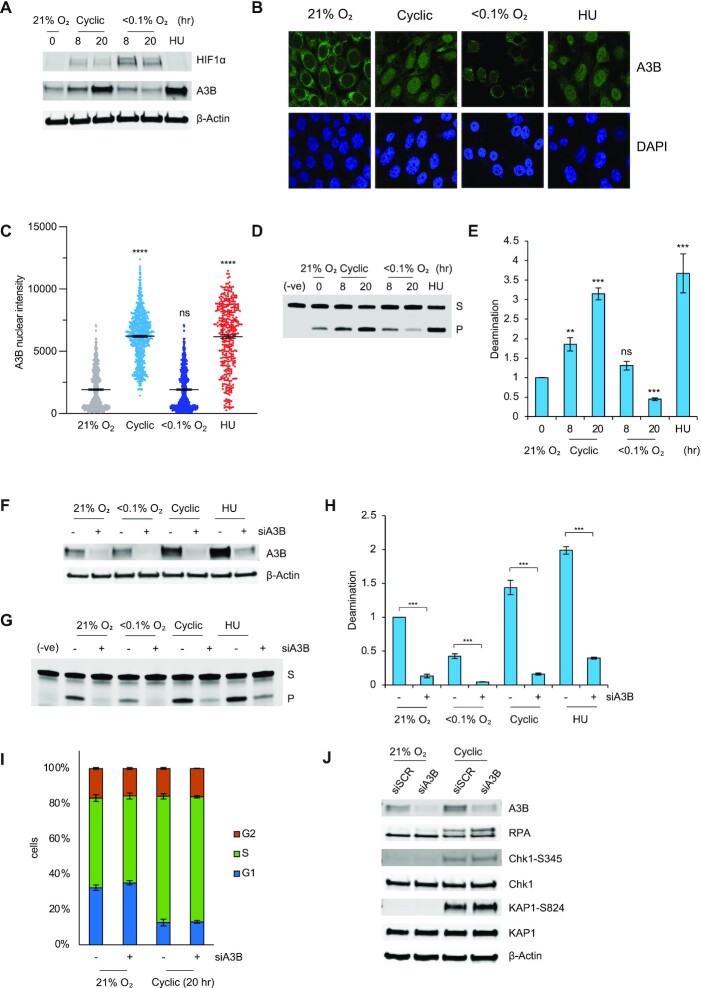
Cyclic hypoxia increases the expression and activity of A3B. (**A**) RKO cells were exposed to the oxygen concentrations indicated for the times shown or HU (2 mM, 20 h). Western blotting was carried out. (**B**) RKO cells were exposed to the indicated oxygen treatments for 20 h or HU (2 mM, 20 h) and co-stained for A3B and DAPI. The cells were visualized by immunofluorescence. (**C**) Quantification of A3B nuclear intensity from part B. (**D**) *In vitro* deamination assay measuring deamination activity in samples collected in the indicated hypoxic treatments for the indicated times or HU (2 mM, 20 h). The top band is the substrate band (S) and the bottom band is the product band (P). (**E**) Quantification of deamination in D. where the average intensity of the product band (P) (bottom) across 3 biological repeats is plotted relative to the intensity of the normoxic product band. (**F**) RKO cells were treated with an siRNA to A3B (siA3B) or a scramble (siSCR) (indicated by –) and then exposed to the hypoxic conditions indicated or HU (2 mM) for 20 h, followed by western blotting. (**G**) The samples from F. were also analyzed by deamination assay. The top band (S) is the substrate band and the bottom band (P) is the product band. (**H**) Quantification of data shown in part G where the average intensity of the product band (P) across 3 biological repeats is plotted relative to the intensity of the normoxic product band. (**I**) RKO cells were treated with siRNA to A3B (siA3B) or a a scramble control (siSCR) followed by exposure to cyclic hypoxia for 20 h. Cells were labeled with BrdU (20 μM) 1 h prior to collection and analyzed by FACS. Quantification of the data shows the percentage of cells in each phase of the cell cycle is shown. Exemplar FACs plots are shown in Figure S4D. (**J**) RKO cells were treated with siRNA to A3B (siA3B), or a scramble control (siSCR) followed by exposure to cyclic hypoxia or normoxia (21% O_2_) for 20 h. Western blotting was carried out. Data from three separate experiments (*n* = 3) are displayed ± standard error of the mean (SEM) unless specified otherwise. ** *P* < 0.01, *** *P* < 0.001.

To determine if cyclic hypoxia would lead to increased APOBEC activity, RKO cells were exposed to either stable (<0.1% O_2_) or cyclic hypoxic conditions and western blotting was carried out for A3B. A 4-fold induction of A3B was seen in the cycling conditions but not stable hypoxia (Figure 5A, Supplementary Figure S4A). Using an antibody previously validated for immunofluorescent staining of A3B (39), we also determine a significant increase in A3B nuclear intensity in response to cyclic hypoxia and HU but not stable hypoxia (Figure 5B, C). Deamination assays demonstrated that, as before, deamination did not increase in stable hypoxia but was significantly increased in response to cyclic hypoxia (Figure 5D, E). We also found that reoxygenating cells to 21% O_2_ after exposure to hypoxia (<0.1% O_2_) lead to increased deamination suggesting that this is not dependent on the specific cyclic conditions used (Supplementary Figure S4B, C). Importantly, we show that the increased deamination in cyclic hypoxia is dependent on A3B as siRNA mediated knock down of A3B abrogated deamination (Figure 5F–H). Previous reports have demonstrated that overexpression of A3B impacts cell cycle distribution as well as the activation of various DDR effector proteins (40,41). In contrast, we found no A3B-dependent changes in the cell cycle in cyclic hypoxia (Figure 5I, Supplementary Figure S4D, E). Furthermore, no change was observed in RPA, Chk1 or KAP1 phosphorylation in cyclic hypoxia when A3B was depleted (Figure 5J). Increased APOBEC expression, including A3B, has been linked to the NF-κB transcription factor, which is also known to be active in hypoxic conditions (42,43). Induction of A3B protein in cyclic hypoxia was partially abrogated by treatment with a NF-κB inhibitor (Bay11-7085) (Supplementary Figure S4F). This finding raised the possibility that cyclic hypoxia-induced cGAS could lead to NF-κB activity and consequent A3B expression (44). However, RKO cells lack STING expression and siRNA mediated depletion of cGAS had no impact on A3B induction (Supplementary Figure S4G) (45).

**Figure 6.**
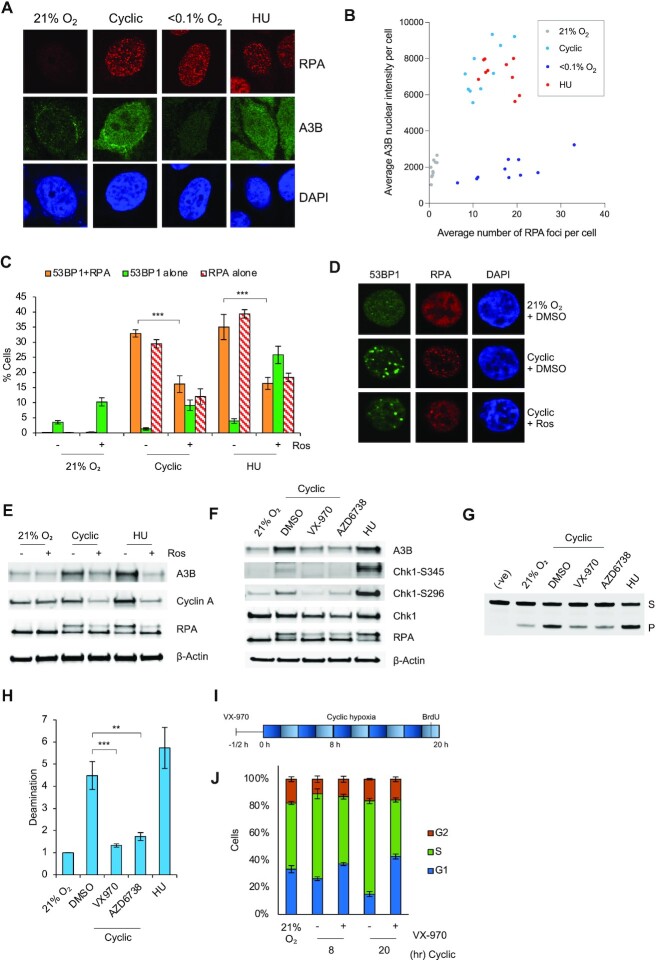
Replication catastrophe leads to increased A3B. (**A**) RKO cells were exposed to the indicated oxygen treatments for 20 h or HU (2 mM, 20 h) and co-stained for RPA, A3B and DAPI. The cells were visualized by immunofluorescence and representative images are shown. (**B**) Quantification of average A3B nuclear intensity and average number of RPA foci per cell. Each dot represents a field of view containing at least 20 cells, a minimum of 200 cells were quantified per condition. (**C**) RKO cells were pretreated with DMSO or roscovitine (Ros) for 30 min (20 μM) and exposed to 21% O_2_, cyclic hypoxia, or HU (2 mM) for 20 h. Cells were stained by immunofluorescence to detect cells with >5 53BP1 and >6 RPA foci and mounted in DAPI. (**D**) Representative images of data shown in C. (**E**) Cells were treated as in C. and western blotting was carried out. (**F**) RKO cells were pre-treated with VX-970 (1 μM), AZD6738 (5 μM), or DMSO for 30 min and then exposed to 20 h cyclic hypoxia or HU alone (2 mM, 20 h). Western blotting was carried out. (**G**) *In vitro* deamination assay on RKO cells pre-treated with VX-970 (1 μM), AZD6738 (5 μM) or DMSO for 30 min and then exposed to 20 h cyclic hypoxia or HU alone (2 mM, 20 h). S = substrate band, P = product band. (**H**) Quantification of the bottom band (P) intensity in samples from part G. (**I**) A schematic showing how cells are treated in part J. (**J**) RKO cells were pre-treated with VX-970 for 30 min (1 μM) or DMSO and then exposed to cyclic hypoxia (8 or 20 h) Cells were labeled with BrdU (20 μM) 1 h prior to collection and analyzed by FACS. Quantification of the percentages of cells in each cycle phase. Data from three separate experiments (*n* = 3) are displayed ± standard error of the mean (SEM) unless specified otherwise. ** *P* < 0.01, *** *P* < 0.001.

To investigate whether the increased A3B seen in cyclic hypoxia was a result of replication catastrophe, we began by co-staining for A3B and RPA. In response to both HU and cyclic hypoxia the nuclear intensity of A3B and the number of nuclear RPA foci increased. In contrast in stable hypoxia, only the number of cells with RPA foci increased (Figure 6A, B). Next, we treated cells with roscovitine to block origin firing. The addition of roscovitine in cyclic hypoxia decreased the number of cells experiencing replication catastrophe as determined by co-staining for 53BP1 and RPA foci (Figure 6C, D, foci count per cell are also shown Supplementary Figure S5A–F). Most importantly and in support of our hypothesis, when roscovitine was used, A3B no longer accumulated in cyclic hypoxia (Figure 6E). In further support of A3B induction depending on origin firing in cyclic hypoxia, addition of the cdc7 inhibitor (PHA-767491), another commonly used method to inhibit origin firing, also blocked A3B accumulation in cyclic hypoxia (Supplementary Figure S5G). These data demonstrate that the expression and activity of A3B is increased in response to cyclic hypoxia and that this activity correlates with replication catastrophe. We hypothesized that the activity of reactive oxygen species (ROS) would also likely contribute to replication catastrophe in cyclic hypoxia and the subsequent A3B activity. Supportive of this hypothesis, treatment with the ROS scavenger N’ acetylcysteine (NAC) in cyclic hypoxia significantly decreased the proportion of cells experiencing replication catastrophe and decreased A3B expression (Supplementary Figure S6A–C, foci numbers per cell are also shown S6D–I).

Having demonstrated that blocking origin firing reduces A3B induction in cyclic hypoxia, we predicted that inhibition of ATR-mediated signaling in cyclic hypoxia would lead to increased origin firing, and therefore could potentially increase A3B accumulation/activity. Cells in cyclic hypoxia were treated with inhibitors to ATR (VX-970, AZD6738) followed by western blotting for A3B. Surprisingly, inhibition of ATR abrogated the induction of A3B in cyclic hypoxia (Figure 6F). This was further confirmed using siRNA to knock down ATR (Supplementary Figure S7A). Treatment with both ATR inhibitors also abrogated APOBEC activity in cyclic hypoxia as assayed by the *in vitro* deamination assay (Figure 6G, H). These data were surprising as they suggest A3B is induced in cyclic hypoxia through an ATR-dependent mechanism, although this is in agreement with the mechanism previously described for HU-induced APOBEC activity (25). However, it was important to consider the impact of ATR inhibition on the cell cycle as replication catastrophe cannot occur outside of S-phase. Cells were exposed to cycling hypoxia in the presence of an ATR inhibitor (VX-970) and the cell cycle analyzed. Inhibition of ATR during cycling conditions prevented the accumulation of cells in S-phase leading instead to an accumulation in the G1 phase (Figure 6I, J, Supplementary Figure S7B). To further support this finding, cells were treated with Chk1 inhibitors (MK-8776 or Gö6976) and exposed to cyclic hypoxia. As observed after ATR inhibition, treatment with the Chk1 inhibitors in cyclic hypoxia led to a G1 accumulation and abrogated the induction of A3B (Supplementary Figure S7C, D). Together, these data suggest that inhibition of ATR/Chk1 in cyclic hypoxia leads to a reduced S-phase population and therefore reduced replication catastrophe and most importantly, link A3B expression/activity with replication catastrophe.

### APOBEC expression and activity correlates with hypoxia in patient samples

**Figure 7.**
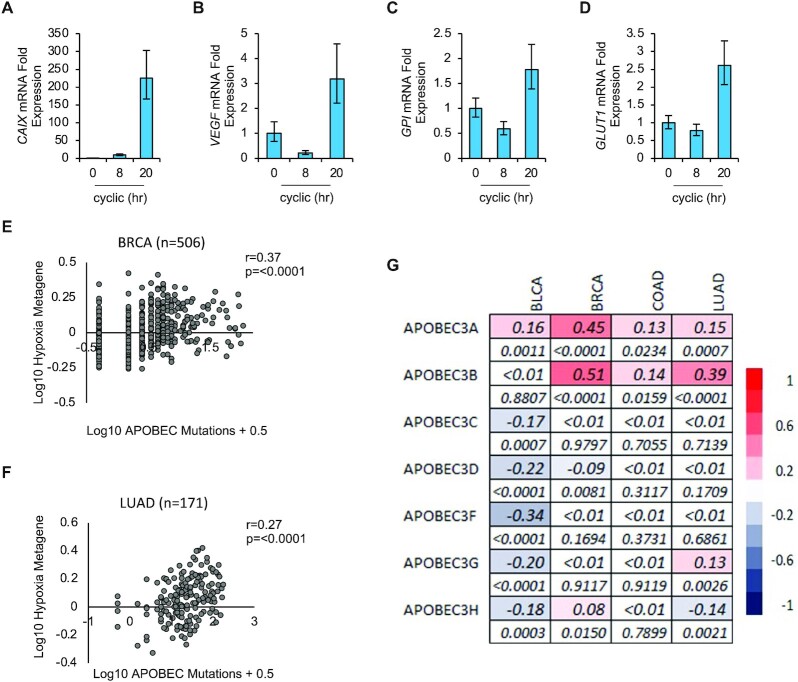
APOBEC activity correlates with hypoxia in patient samples. (**A–D**) RKO cells were exposed to cyclic hypoxia (20 h) followed by qPCR analysis for the hypoxia-inducible genes indicated (CAIX, VEGF, GPI and GLUT1). *n* = 1. (**E, F**) Correlation between the hypoxia metagene and the number of APOBEC mutations in the two TCGA datasets where mutational data were available for >100 cases. APOBEC mutations refers to the number of tCw to tTw and tCw to tGw changes. The log mean expression of the hypoxia signature for each cancer dataset is shown compared to the scaled number of APOBEC mutations (see methods). The Spearman's rank-order correlation coefficient (rho), and p value are displayed. (**G**) Spearman's rank-order correlation between the hypoxia metagene and APOBEC mRNA expression in TCGA datasets, correlation and significance are shown. For each gene the correlation values (top raw) and *P* values (bottom raw) are given. Color scale of correlation values is shown on the right of the heatmap.

Finally, we sought to identify a link between tumor hypoxia and APOBEC expression/activity in patient samples. We have shown that hypoxic conditions which include cyclic variations between <0.1% and 2% O_2_ lead to the induction of A3B expression and activity. Cyclic hypoxia is a physiologically relevant condition, although detailed study of this phenomenon has been hampered by difficulties in measuring the periodicity in patients. As of yet, there are no clinically useful markers of cyclic hypoxia in routine use (6,46,47). Therefore, we used a validated hypoxia signature, which is primarily made up of HIF target genes to determine a link between hypoxia and A3B expression in patient tumors (29). Our rationale to support the use of the hypoxia signature is that cells experiencing fluctuating/cyclic hypoxic conditions also stabilize HIF1α (Figure 5A) and therefore expression of associated HIF-target genes will be increased. To support this rationale, we verified that genes included in the hypoxia signature were induced in response to cyclic hypoxia (Figure 7A-D). Next, using the TCGA patient cohort, and the cancer types where there were more than 100 samples with available APOBEC mutational data (BRCA and LUAD), we found a significant correlation between the number of APOBEC mutations present and the hypoxic signature in both datasets (Figure 7E, F). In further support of our conclusions, we found a significant positive correlation between the hypoxia signature and the expression of A3A and A3B in all datasets for A3A, and three out of four datasets for A3B, but not the other A3s (Figure 7G).

## DISCUSSION

We have demonstrated that the biological response to replication stress is determined by the level of accompanying DNA damage (replication catastrophe). Exposure to hypoxia (<0.1% O_2_) induces significant replication stress but this occurs in the apparent absence of DNA damage and does not lead to APOBEC expression/activity. In contrast, replication stress induced by either HU or cycling hypoxia leads to replication catastrophe and results in APOBEC expression/activity. The significance of our finding is highlighted by evidence suggesting that cycling hypoxia leads to increased APOBEC activity in patient samples.

Though it has yet to be measured routinely in human tumors, a wealth of evidence from *in vivo* experiments suggest that cyclic hypoxia is commonplace in solid tumors (5,46,48–51). Though much study has been done to date on the effects of cyclic hypoxia on treatment modalities such as radiotherapy and chemotherapy, this has seldomly been done at oxygen concentrations that have been detected *in vivo*. The vast majority of studies utilize ambient air (21% O_2_) as the reoxygenation portion of their cyclic schedule (52), while the maximum oxygen concentration predicted to occur in xenografts is closer to 5% O_2_ (5). Our cyclic hypoxia schedule represents a more physiological relevant condition and the first description of an endogenous source of replication catastrophe that likely occurs in human tumors. Therefore, the link between replication stress and APOBEC expression/activity is a plausible mechanism by which the APOBEC signature can manifest in human cancer. Notably, we found that the significance of the correlations between A3B and the hypoxia metagene differs between the cancer types we investigated. However, this is not surprising given that the presence of the APOBEC-mediated mutational signature has also been shown to be cancer type specific (20,26).

Our study showed an upregulation of A3D, and A3H and to a lesser extent A3C and A3F in stable hypoxia (<0.1% O_2_). As APOBECs can also bind RNA as a substrate, mRNA editing has been proposed as a possible function of the family. Recently, a limited number of studies have implicated both A3A and A3G in mRNA editing (53–55). Interestingly, while there was overlap between the transcripts edited by A3A and A3G, many were specifically edited by one or the other. These observations raise two interesting questions; Firstly, whether the APOBECs upregulated in hypoxia can contribute to gene regulation through mRNA editing and secondly, if so, do they influence overlapping or distinct sets of genes? Furthermore, A3A was shown to edit the mRNA of a specific gene, succinate dehydrogenase B (SDHB), and this activity increased in response to mild levels of hypoxia (1% O_2_) (55). Further study would be required to determine whether hypoxic regulation of APOBEC mRNA editing by A3A is a global phenomenon or restricted to specific genes such as SDHB.

A number of previous reports have described increased replication stress signaling and a G2/M phase cell cycle arrest following A3B overexpression (40,41). In contrast, we did not find an A3B-dependent effect on either replication stress or cell cycle profile. A notable difference between the previous studies and ours is the method of A3B induction, cyclic hypoxia induces the endogenous expression of A3B whereas previous reports rely on levels of A3B resulting from exogenous overexpression. Differences in the overall quantity and localization of A3B in the cell could potentially explain the discrepancy. Interestingly, the effects of both endogenously interferon-stimulated A3A and ectopically expressed A3A on cell viability has been investigated and found that while ectopically expressed A3A significantly decreased cell viability, endogenously expressed A3A did not (56).

A limitation of our study is that A3A expression could not be detected in the RKO cell line which is significant as the generation of the APOBEC mutational signature in human cancers has been attributed to A3B and A3A (20–24). However, A3A expression is generally restricted to immune cells and is seldomly detected in cancer cell lines derived from other tissues (26,57,58). Further study in alternative models would be required to determine the effect of hypoxia on A3A expression/activity. It is possible that the increases in A3B-mediated deamination we have observed in cyclic hypoxia would be further increased in cells also expressing A3A.

Further investigation is required to determine the fate of cells that experience replication catastrophe due to cyclic hypoxia. Interestingly, our data clearly demonstrate that in contrast to stable hypoxia (<0.1% O_2_), cyclic hypoxia-induced p53 does not lead to apoptosis. Our findings demonstrating an increase in cell size and a G2 cell cycle arrest (Figures 3G–J and 4E, F), as well as previous reports investigating replication catastrophe, suggest that senescence is a possible outcome (12). However, it is important to note that our study was carried out in a p53 proficient cell line, which we predict will significantly impact the cellular response to cyclic hypoxia. It is plausible that in the presence of wild type p53, cyclic hypoxia contributes to tumourigenesis through senescence. However, in the absence of p53 cells which experience non-lethal levels of cyclic hypoxia-induced replication catastrophe/DNA damage could increase genomic instability through elevated APOBEC expression/activity.

Targeting APOBECs is an emerging clinical strategy. Decreasing APOBEC expression could be used as an enhanced treatment strategy to limit tumorigenesis and the development of treatment resistance through a decreased mutation rate. Supportively, a study investigating the development of tamoxifen resistance *in vivo* showed a significant A3B dependent effect on the speed at which tumors developed treatment resistance (59). Strategies to inhibit APOBEC activity include, the development of a modified oligonucleotide that can be used to selectively inhibit A3B *in vitro* (60). The development of inhibitory compounds and preclinical testing will be required to determine if these strategies will prove efficacious in a clinical setting. Somewhat controversially, it is worth considering that increasing APOBEC activity in cancers could be beneficial and particularly in the immunosuppressive hypoxic regions of tumors. Increasing APOBEC expression has been shown to increase the efficacy of immune checkpoint blockade therapy due to increased neoepitope presentation (27). Overall, our study provides the first mechanistic insight into the mechanism of increased APOBEC activity and links this mechanism to the presence of APOBEC-mediated mutations in human cancers.

## DATA AVAILABILITY

All data is available from the corresponding author upon request.

## Supplementary Material

gkab551_Supplemental_Files
