# Supplementary material for: Replication catastrophe induced by cyclic hypoxia leads to increased APOBEC3B activity
Source: Nucleic Acids Res. 2021 Jul 1;49(13):7492–506. doi: 10.1093/nar/gkab551 (PMC8287932; doi:10.1093/nar/gkab551)
Supplement: gkab551_Supplemental_Files [file gkab551_supplemental_files.zip › Bader et al. Complete SI.pdf]

Replication catastrophe induced by cyclic hypoxia leads to increased APOBEC3B activity

Samuel Bader, Tiffany S. Ma, Charlotte J. Simpson, Jiachen Liang, Sakura Eri B. Maezono, Monica M. Olcina, Francesca M. Buffa and Ester M. Hammond*

Oxford Institute for Radiation Oncology, Department of Oncology, The University of Oxford, Oxford, OX3 7DQ, UK

Contents page

Table S1 - qRT-PCR primer sequences 2

SI Methods 2

SI Figure legends 3

Table S1 – Sequences of primers used for qRT-PCR

Gene	Oligonucleotide name	Sequence	Working concentration	Source
18S	18S F5<br>18S R5	TAGAGGGACAAGTGGCGTTC<br>CGGACATCTAAGGGCATCAC	5 μM	Primer Blast
A3A	A3A_F1<br>A3A_R1	GGCCCAGACACTTGATGGAT<br>TGTAGAAAGCCCCTGTGCTG	1 μM	Primer Blast
A3A	A3A_F2<br>A3A_R2	CAGCACAGGGGCTTTCTACA<br>TTCTCCTGAAGGAACGCACG	1 μM	Primer Blast
A3A	A3A_F3<br>A3A_R3	GAAGGGACAAGCACATGGAAGC<br>ATCTACTTGATCGGGAGCATAC	1 μM	Primer Blast
A3B	A3B_F2<br>A3B_R2	TCTATGGTCGGAGCTACACTTG<br>GCACATTTCTGCGTGGTACTG	1 μM	Primer Blast
A3C	A3C_F1<br>A3C_R1	CAAGGACGCTGTAAGCAGGA<br>TGCCTTCATCGGGTTTCTGA	2 μM	Primer Blast
A3D	A3D_F2<br>A3D_R1	GATAGAGATTGGCGGTGGGT<br>CAGTATGCAAAGTCTTCATAGTCCA	2 μM	Primer Blast
A3F	A3F_F1<br>A3F_R1	CCGTTTGGACGCAAAGAT<br>CCAGGTGATCTGGAAACACTT	2 μM	Primer Blast
A3G	A3G_F1<br>A3G_R1	CCGAGGACCCGAAGGTTAC<br>TCCAACAGTGCTGAAATTCG	1 μM	Primer Blast
A3G	A3G_F2<br>A3G_R2	ACCCTGACCATCTTTGTTGC<br>TCCAACAGTGCTGAAATTCG	1 μM	Primer Blast
A3G	A3G_F3<br>A3G_R3	GCATCGTGACCAGGAGTATG<br>TCCAACAGTGCTGAAATTCG	1 μM	Primer Blast
A3H	A3H_F1<br>A3H_R1	GCCAGAAGCACAGATCAGAAAC<br>GCGTCAGCTGGTAACACAAG	5 μM	Primer Blast
INPP5D	INPP5D_F1<br>INPP5D_R1	GAGAGGAGGGAGCAGAAGGT<br>GCTTTCTGCTTGGTGTAGGC	1 μM	Primer Blast
GLUT1	GLUT1_F1<br>GLUT1_R1	ATACTCATGACCATCGCGCTAG<br>AAAGAAGGCCACAAAGCCAAAG	4 μM	Primer Blast
CHOP	CHOP_F1<br>CHOP_R1	GGAGCATCAGTCCCCCACTT<br>TGTGGGATTGAGGGTCACA	1 μM	Primer Blast

SI Methods

Quantification of 53BP1/RPA foci per cell

Quantification of 53BP1/RPA foci was achieved using image J. Briefly, a threshold value for what intensity constitutes a foci was selected based on an analysis of the foci present in the positive control. The macro then detects cells in the provided image based on the DAPI staining and according to set cell size parameters. The number of foci within each cell that are greater than the foci value set are then counted as foci per cell. Using this approach, the 53BP1 and RPA foci within the same cell were quantified.

SI Figure legends

Figure S1. APOBEC expression is altered in an oxygen-dependent manner

A-D. RKO cells were treated with the indicated oxygen concentration or HU (2 mM) for 20 hr. The mRNA levels of *INPP5D* (A), *CHOP* (B), *A3C* (C), and *A3F* (D) as a relative fold change to normoxia (21% O_2) are shown. n=2

E. MCF7 cells were treated with <0.1% O_2 for the indicated times. The mRNA levels of *A3H* as a relative fold change to normoxia (21% O_2) are shown.

F, G. $RKO^{HIF1a+/+}$ and $RKO^{HIF1a-/-}$ were treated with <0.1% O_2 for 24 hr and RT-qPCR was carried out for *A3D* (F) and *A3H* (G) mRNA.

H. RKO cells were treated with siRNA to p53 (sip53) or a scramble control (siSCR) followed by hypoxia (<0.1% O_2) for 20 hr. RT-qPCR was carried out for *INPP5D* mRNA.

I. The indicated cell lines were treated with <0.1% O_2 or normoxia for 20 hr before RT-qPCR was carried out for *A3B* mRNA expression (n=1).

J. The p53 status of the cell lines in G. are indicated.

K. $HCT116^{p53+/+}$ and $HCT116^{p53-/-}$ cells were exposed to hypoxia (<0.1% O_2) for the times indicated or HU (2 mM, 20 hr) followed by western blotting.

Data from three separate experiments (n=3) are displayed ± standard error of the mean (SEM) unless specified otherwise. * = p = <0.05, ** = p = <0.01, *** = p = <0.001, ns= non-significant.

Figure S2. Cell cycle analysis of RKO cells exposed to hypoxia

A. Quantification of the data shown in Figure 2A showing the percentages of cells in each phase of the cell cycle.

B. The percentage of RKO cells treated with the indicated oxygen concentration or HU (2 mM) for 20 hr in the sub G1 cell cycle phase. FACS plots can be seen in Figure 2A.

C. RKO cells were pretreated with DMSO or roscovitine (Ros) (20 μM) for 30 min and exposed to the indicated oxygen concentration or HU (2 mM) for 20 hr. Cells were labeled with BrdU (20 μM) 1 hr prior to collection and analyzed by FACS. A quantification of this data can be found in Figure 2C.

D, E, F. The data shown in Figure 2D were analyzed to determine the total number of 53BP1 and RPA foci in each of the individual cells imaged.

Data from three separate experiments (n=3) are displayed ± standard error of the mean (SEM). *** = p = <0.001.

Figure S3. Cyclic hypoxia induces replication stress

A. A representative readout of the oxygen percentage over the course of an experiment from the built-in oxygen probe contained within the Don Whitley M35 variable atmosphere workstation.

B. Quantification of data in Figure 3C, D. The number of foci in every cell that had experienced replication stress (>6 RPA foci) under each treatment condition is shown. Black bars represent the mean number of RPA foci.

C. RKO cells were exposed to the oxygen concentrations indicated for the times shown. Western blotting was carried out. n=2

D. RKO cells were exposed to 8 or 20 hr of cyclic hypoxia and co-stained for γH2AX, RPA-S4/S8 and DAPI. The cells were visualized by immunofluorescence and the quantification of nuclear intensities of γH2AX and RPA-S4/S8 per cell is shown. The square of correlation (R^2) values for both conditions are shown.

E. RKO cells were exposed to 8 or 20 hr of cyclic hypoxia and incubated with EdU (10 μM) during the last hour of hypoxia treatment. The cells were co-stained for EdU and 53BP1 and

visualized by immunofluorescence. The percentage of cells with >5 53BP1 per nucleus in EdU positive and EdU negative cells are shown.

F-I. The data shown in Figure 4C were analyzed to determine the total number of 53BP1 and RPA foci in each of the individual cells imaged. The 21% O_2 and HU treatment conditions are the same as shown in Figure S2D, F as these experiments were conducted at the same time. Data from three separate experiments (n=3) are displayed unless otherwise indicated ± standard error of the mean (SEM). *** = p = <0.001, **** = p <0.0001 ns= non-significant.

Figure S4. A3B expressions increases in response to cyclic hypoxia.

A. Quantification of western blots shown in Figure 5A. Data show mean ± SEM from n=3 biological experiments.

B. RKO cells were exposed to the indicated oxygen concentrations or HU (2 mM, 24 hr) followed by a deamination assay. Reox = 6 hr of <0.1% O_2 followed by 24 hr of 21% O_2. The top band (S) is the substrate band and the bottom band (P) is the product band. n=1

C. Western blotting was carried out on samples treated as in B.

D. Representative FACS plots of data shown in Figure 5I. Transfection agent alone (mock) controls are also shown.

E. Confirmation of successful A3B knockdown for data shown in Figure 5I by western blot. Transfection agent alone (mock) controls are also shown.

F. RKO cells were treated with the indicated concentrations of Bay11-7085 and cyclic hypoxia for 20 hr. Western blotting was carried out; KAP1 is used as a loading control. n=2

G. RKO cells were treated with siRNA cGAS (sicGAS) or siRNA scramble (siSCR) (indicated by -) and exposed to cyclic hypoxia, normoxia (21% O_2), or HU (2 mM) for 20 hr. Western blotting was carried out.

Data from three separate experiments (n=3) are displayed ± standard error of the mean (SEM) unless specified otherwise. ** = p = <0.01, *** = p = <0.001.

Figure S5. Mechanism of A3B induction in cyclic hypoxia.

A-F. The data shown in Figure 6C were analyzed to determine the total number of 53BP1 and RPA foci in each of the individual cells imaged.

G. RKO cells were pretreated with DMSO or cdc7i (20 μM) for 30 min and exposed to 21% O_2, cyclic hypoxia, or HU (2 mM) for 20 hr. Western blotting was carried out. n=2

Figure S6. Mechanism of A3B induction in cyclic hypoxia.

A. RKO cells were pretreated with DMSO or NAC (5 mM) for 30 min and exposed to 21% O_2, cyclic hypoxia, or HU (2 mM) for 20 hr. Cells were stained by immunofluorescence to detect cells with >5 53BP1 and >6 RPA foci

B. Representative images from part A are shown.

C. RKO cells were treated as in part A. followed by western blotting for A3B and β-actin.

D-I. The data shown in Figure S6A were analyzed to determine the total number of 53BP1 and RPA foci in each of the individual cells imaged. The three DMSO treated conditions are the same as shown in Figure S5A-C as these experiments were conducted at the same time.

Data from three separate experiments (n=3) are displayed ± standard error of the mean (SEM) unless specified otherwise. ** = p = <0.01, ns= non-significant.

Figure S7. Mechanism of A3B induction in cyclic hypoxia.

A. RKO cells were treated with siRNA to ATR (siATR) or a scramble control (siSCR) (indicated as -) and exposed to the indicated oxygen concentrations for 20 hr. Western blotting was carried out. n=2

B. Representative FACS plots of data shown in Figure 6J.

C. RKO cells were pre-treated with DMSO, VX-970 (1 μM), AZD6738 (10 μM), (Gö6976 1 μM), or (MK-8776 1 μM) for 30 min and exposed to normoxia (21% O_2) or cyclic hypoxia for 20 hr. Cells were pulsed with BrdU (20 μM) 1 hr prior to collection and analyzed by FACS. The percentage of cells in S-phase in each plot is shown in the top right corner. A schematic showing how cells are treated is shown above the FACS plots.

D. RKO cells were treated as in C. or with HU (2mM, 20 hr) and western blotting was carried out as indicated.

Data from three separate experiments (n=3) are displayed ± standard error of the mean (SEM) unless specified otherwise. ** = p = <0.01, ns= non-significant.

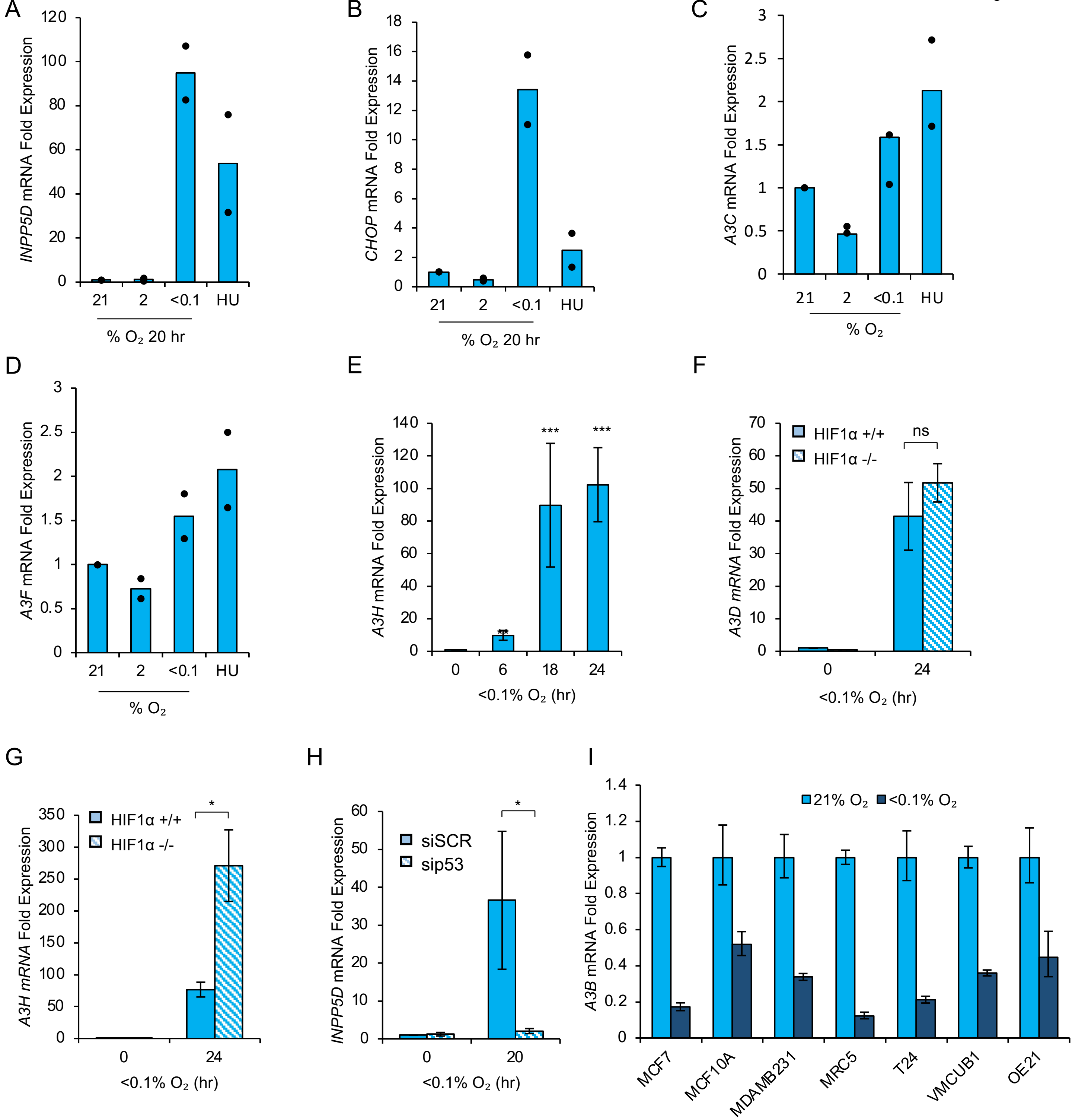

J

Cell line	p53 status
MCF7	wt
MCF10A	wt
MDAMB231	missense
MRC5	wt
T24	nonsense
VMCUB1	missense
OE21	deletion

K

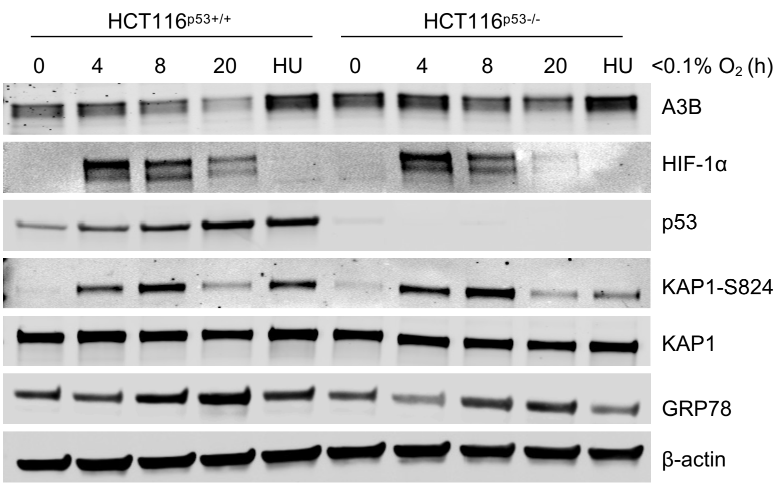

Figure S2

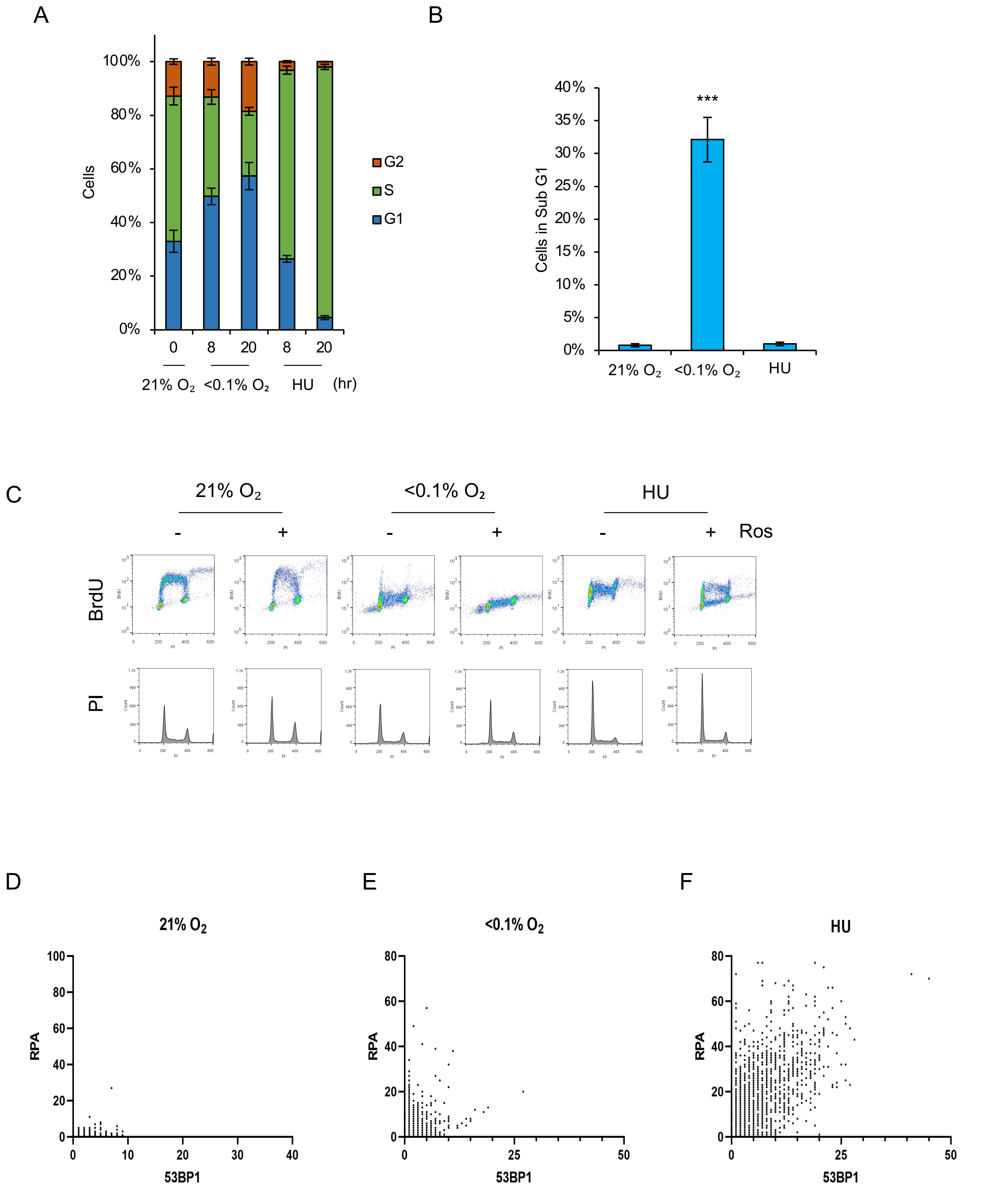
A
100%
80%
60%
40%
20%
0%
Cells
G2
S
G1
0
8
20
8
20
21% O2
<0.1% O2
HU
(hr)
B
40%
35%
30%
25%
20%
15%
10%
5%
0%
Cells in Sub G1
***
21% O2
<0.1% O2
HU
C
21% O2
<0.1% O2
HU
-
+
-
+
-
+
Ros
BrdU
PI
D
21% O2
RPA
53BP1
E
<0.1% O2
RPA
53BP1
F
HU
RPA
53BP1

Figure S3

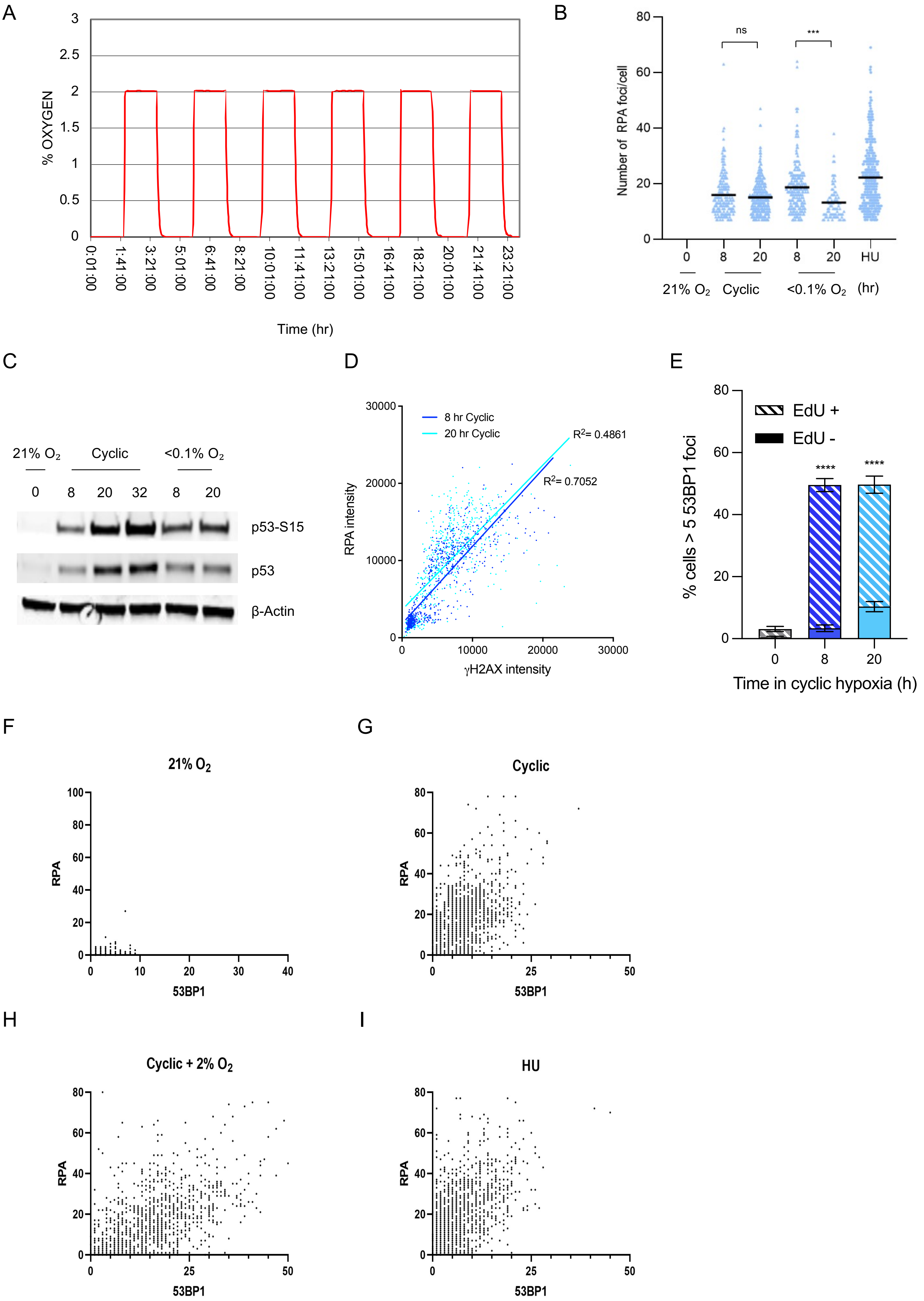

A
% OXYGEN
3
2.5
2
1.5
1
0.5
0
0:01:00
1:41:00
3:21:00
5:01:00
6:41:00
8:21:00
10:01:00
11:41:00
13:21:00
15:01:00
16:41:00
18:21:00
20:01:00
21:41:00
23:21:00
Time (hr)
B
Number of RPA foci/cell
80
60
40
20
0
ns
***
0
8
20
8
20
HU
21% O2
Cyclic
<0.1% O2
(hr)
C
21% O2
Cyclic
<0.1% O2
0
8
20
32
8
20
p53-S15
p53
β-Actin
D
30000
20000
10000
0
8 hr Cyclic
20 hr Cyclic
R2= 0.4861
R2= 0.7052
RPA intensity
γH2AX intensity
E
80
60
40
20
0
EdU +
EdU -
****
****
% cells > 5 53BP1 foci
Time in cyclic hypoxia (h)
F
21% O2
100
80
60
40
20
0
RPA
10
30
40
53BP1
G
Cyclic
25
50
53BP1
RPA
H
Cyclic + 2% O2
RPA
53BP1
I
HU
RPA
53BP1

Figure S4

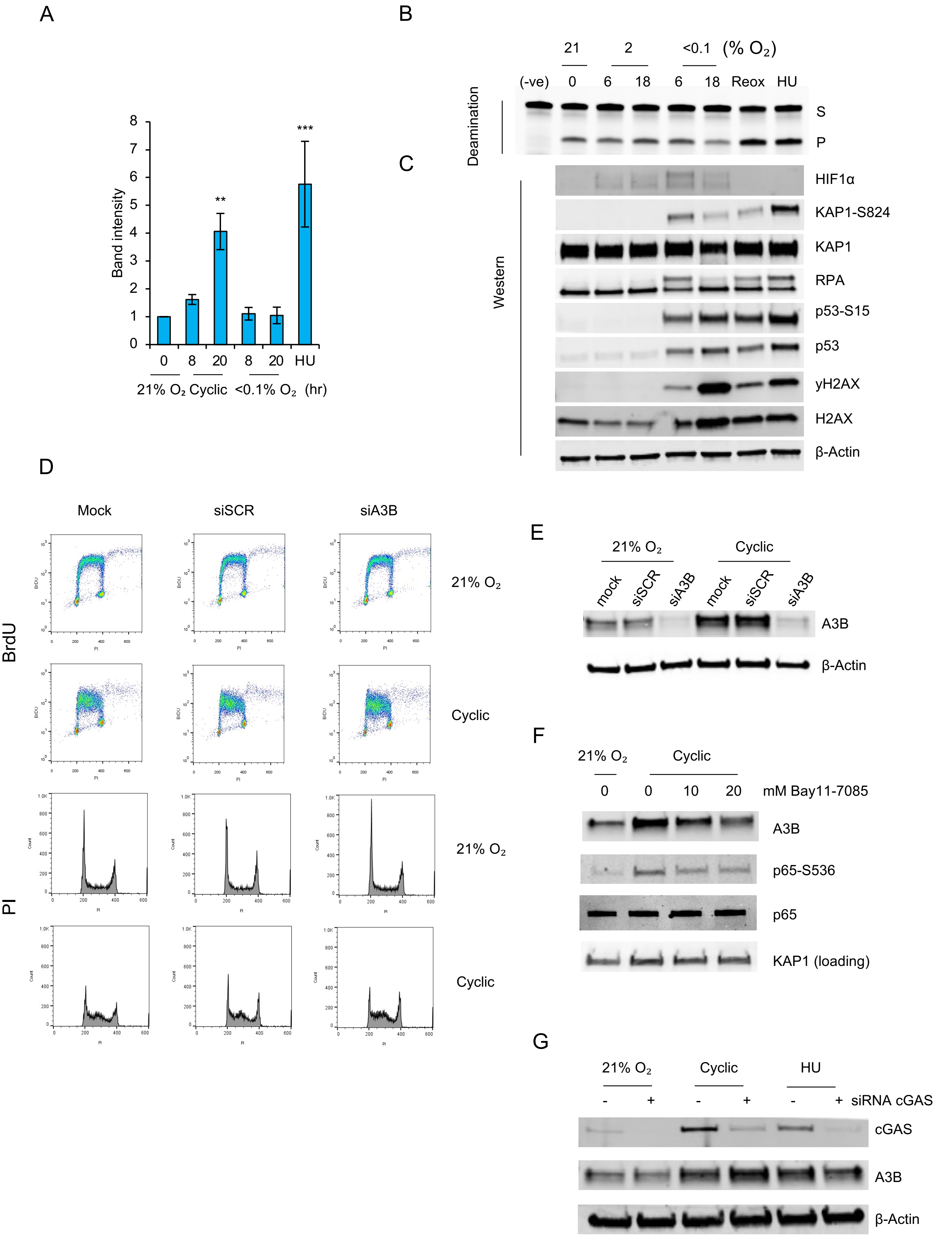

A
Band intensity
8
7
6
5
4
3
2
1
0
**
***
0
8
20
8
20
HU
21% O_2 Cyclic <0.1% O_2 (hr)
B
C
21
2
<0.1
(% O_2)
(-ve)
0
6
18
6
18
Reox
HU
Deamination
S
P
Western
HIF1α
KAP1-S824
KAP1
RPA
p53-S15
p53
yH2AX
H2AX
β-Actin
D
Mock
siSCR
siA3B
BrdU
PI
21% O_2
Cyclic
21% O_2
Cyclic
E
21% O_2
Cyclic
mock
siSCR
siA3B
mock
siSCR
siA3B
A3B
β-Actin
F
21% O_2
Cyclic
0
0
10
20
mM Bay11-7085
A3B
p65-S536
p65
KAP1 (loading)
G
21% O_2
Cyclic
HU
-
+
-
+
-
+
siRNA cGAS
cGAS
A3B
β-Actin

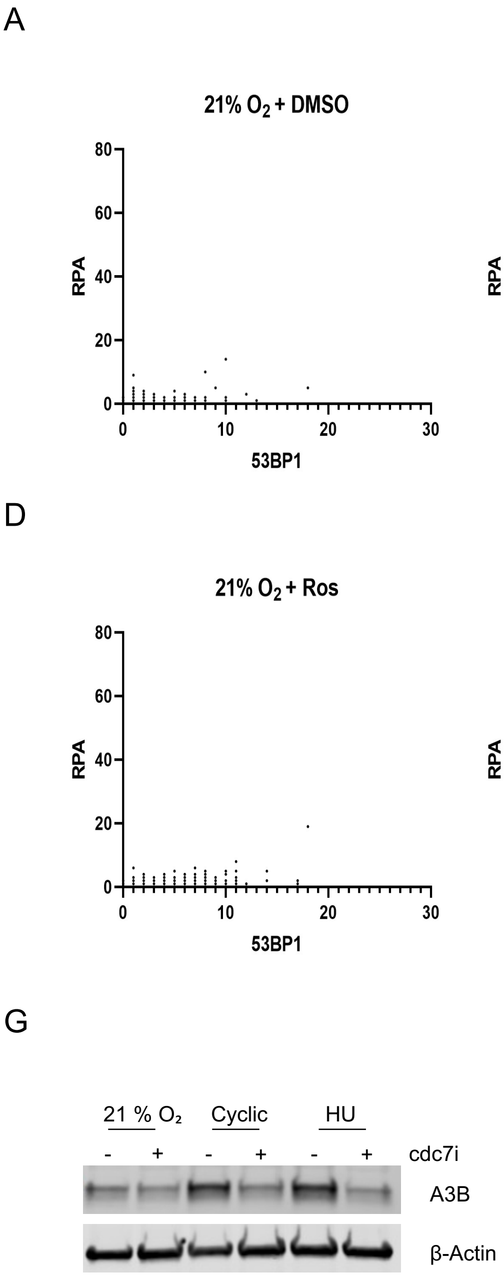
A
21% O2 + DMSO
RPA
53BP1
B
Cyclic + DMSO
RPA
53BP1
C
HU + DMSO
RPA
53BP1
D
21% O2 + Ros
RPA
53BP1
E
Cyclic + Ros
RPA
53BP1
F
HU + Ros
RPA
53BP1
G
21 % O₂
Cyclic
HU
cdc7i
A3B
β-Actin

Figure S6

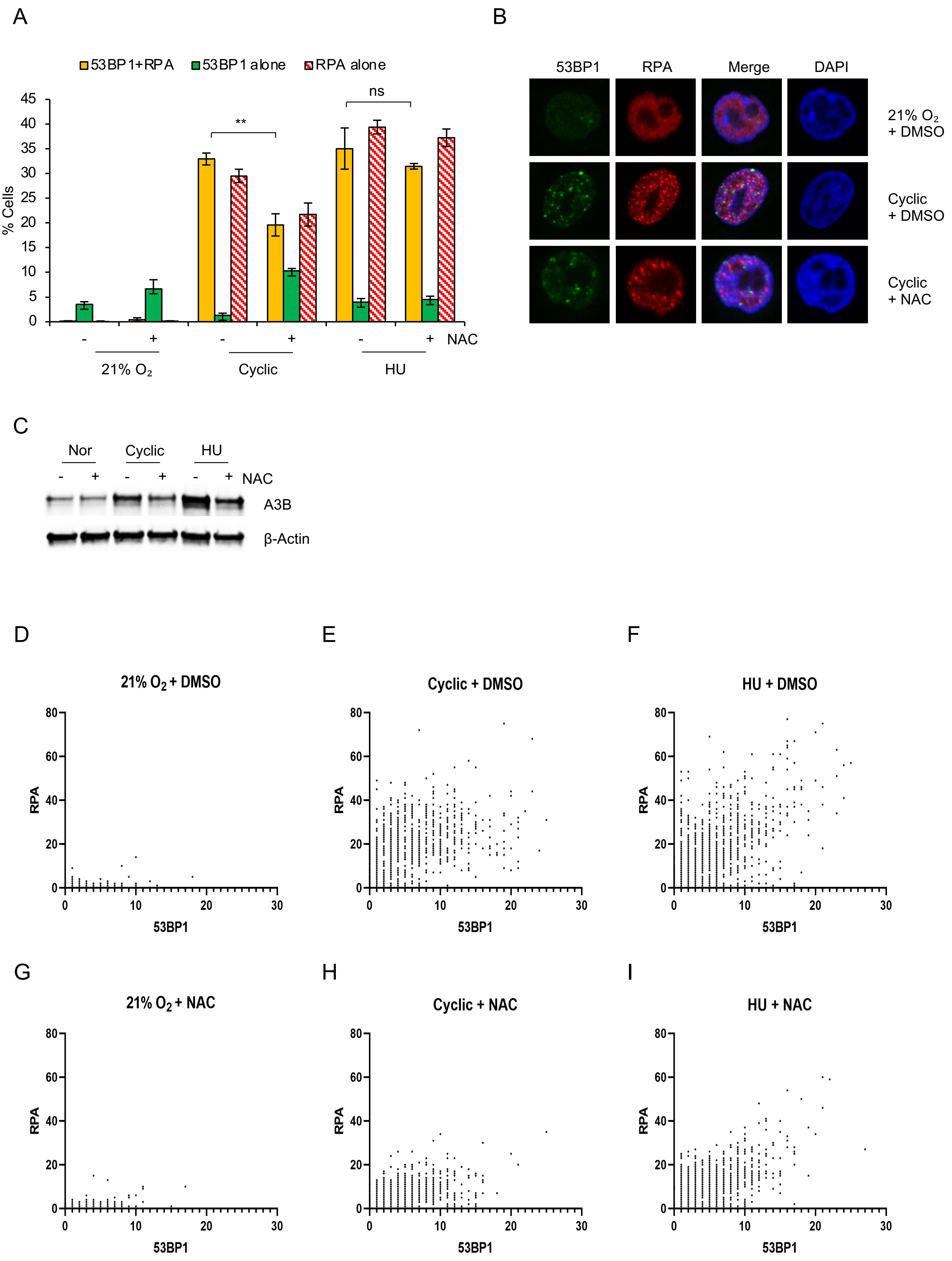
A
53BP1+RPA
53BP1 alone
RPA alone
% Cells
**
ns
NAC
21% O2
Cyclic
HU
B
53BP1
RPA
Merge
DAPI
21% O2 + DMSO
Cyclic + DMSO
Cyclic + NAC
C
Nor
Cyclic
HU
NAC
A3B
β-Actin
D
21% O2 + DMSO
E
Cyclic + DMSO
F
HU + DMSO
G
21% O2 + NAC
H
Cyclic + NAC
I
HU + NAC
RPA
53BP1

Figure S7

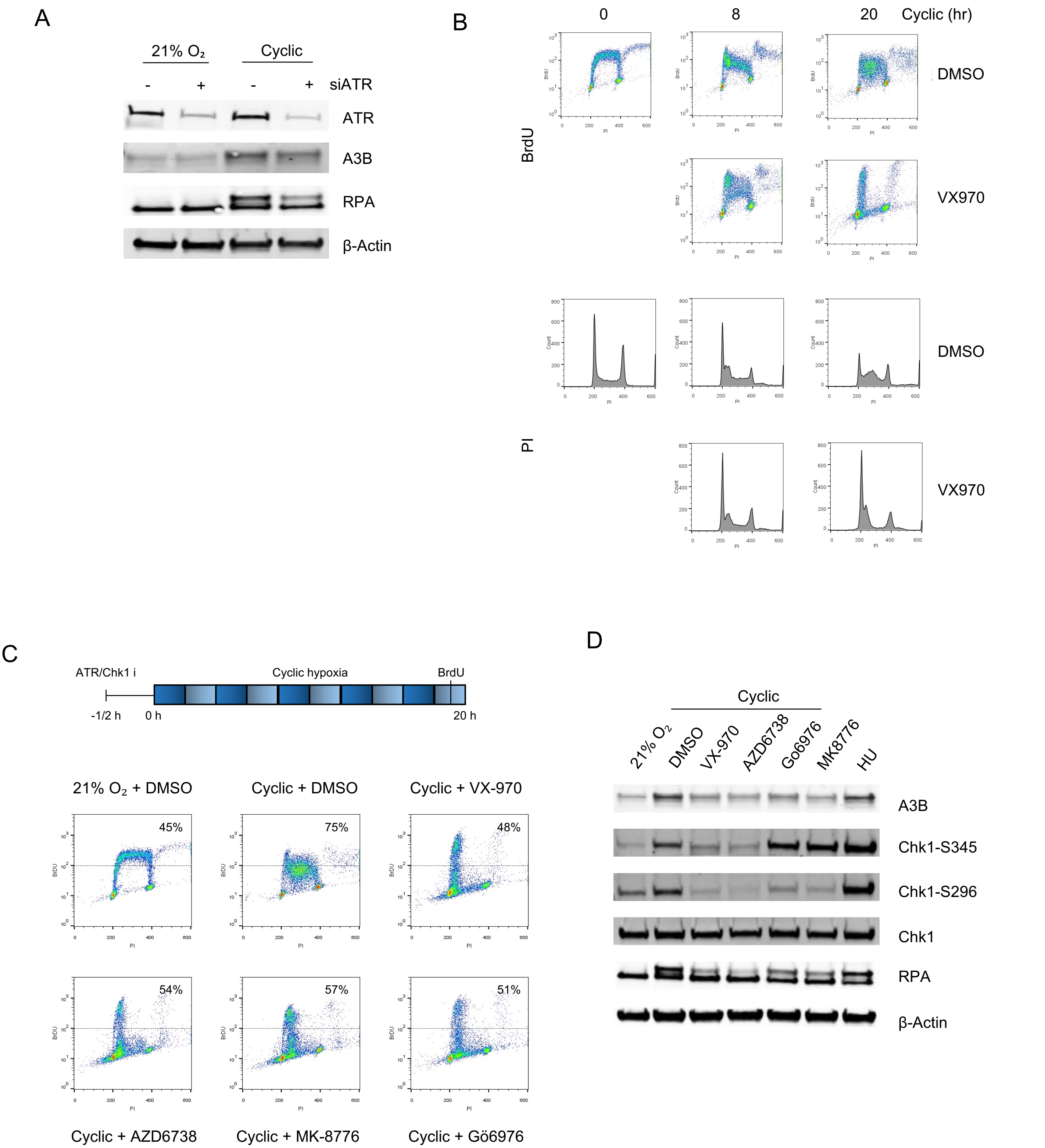

A
21% O2
Cyclic
- + - + siATR
ATR
A3B
RPA
β-Actin
B
0
8
20
Cyclic (hr)
BrdU
DMSO
VX970
PI
DMSO
VX970
C
ATR/Chk1 i
Cyclic hypoxia
BrdU
-1/2 h
0 h
20 h
21% O2 + DMSO
Cyclic + DMSO
Cyclic + VX-970
45%
75%
48%
54%
57%
51%
Cyclic + AZD6738
Cyclic + MK-8776
Cyclic + Gö6976
D
Cyclic
21% O2
DMSO
VX-970
AZD6738
Go6976
MK8776
HU
A3B
Chk1-S345
Chk1-S296
Chk1
RPA
β-Actin
